# Ablation of glucosinolate accumulation in the oil crop *Camelina sativa* by targeted mutagenesis of genes encoding the transporters GTR1 and GTR2 and regulators of biosynthesis MYB28 and MYB29

**DOI:** 10.1111/pbi.13936

**Published:** 2022-10-14

**Authors:** Georg Hölzl, Barno Ruzimurodovna Rezaeva, Jochen Kumlehn, Peter Dörmann

**Affiliations:** ^1^ Institute of Molecular Physiology and Biotechnology of Plants University of Bonn Bonn Germany; ^2^ Plant Reproductive Biology Leibniz Institute of Plant Genetics and Crop Plant Research (IPK) Gatersleben Seeland Germany

**Keywords:** *Camelina sativa*, glucosinolate, oil, lipid, protein

## Abstract

*Camelina sativa* is an oil crop with low input costs and resistance to abiotic and biotic stresses. The presence of glucosinolates, plant metabolites with adverse health effects, restricts the use of camelina for human and animal nutrition. Cas9 endonuclease‐based targeted mutagenesis of the three homeologs of each of the glucosinolate transporters *CsGTR1* and *CsGTR2* caused a strong decrease in glucosinolate amounts, highlighting the power of this approach for inactivating multiple genes in a hexaploid crop. Mutagenesis of the three homeologs of each of the transcription factors CsMYB28 and CsMYB29 resulted in the complete loss of glucosinolates, representing the first glucosinolate‐free Brassicaceae crop. The oil and protein contents and the fatty acid composition of the *csgtr1csgtr2* and *csmyb28csmyb29* mutant seeds were not affected. The decrease and elimination of glucosinolates improves the quality of the oil and press cake of camelina, which thus complies with international standards regulating glucosinolate levels for human consumption and animal feeding.

## Introduction

*Camelina sativa* has gained increasing interest as sustainable oil crop for food and non‐food applications (Pilgeram *et al*., 2007). Camelina is grown in Spain, France, Italy, Eastern Europe as well as the United States and Canada, often to produce organic oils but also as a feedstock or as renewable resource (Alberghini *et al*., 2022; Mondor and Hernández‐Álvarez, 2022). Like rapeseed (*Brassica napus*) and the model plant *Arabidopsis thaliana*, camelina is a member of the Brassicaceae family. The oil content of camelina seeds is in the range of 30%–49% with a high proportion (30%–43%) of the essential ω‐3 fatty acid α‐linolenic acid, while the amount of the undesired erucic acid is below 3% (Vollmann and Eynck, 2015). The protein‐rich press cake obtained after oil extraction is employed for animal feeding (Pilgeram *et al*., 2007; Zubr, 1997). However, the presence of glucosinolates can represent a limitation for the production of livestock feed. Camelina seeds contain three aliphatic glucosinolates, glucoarabin (9‐methyl‐sulfinyl‐nonyl‐glucosinolate, GL9), glucocamelinin (10‐methyl‐sulfinyl‐decyl‐glucosinolate, GL10) and gluconesliapaniculatin (11‐methyl‐sulfinyl‐undecyl‐glucosinolate, GL11; Berhow *et al*., 2013). Glucosinolates and their toxic breakdown products can limit the value of oil and the press cake (European Food Safety Authority (EFSA), 2008; Matthäus and Zubr, 2000). The adverse effects of glucosinolates include growth retardation, reduced performance and impairment of kidney and liver functions of the livestock. The glucosinolate content in camelina meal after solvent extraction can be further decreased during post‐processing and can thus meet the regulatory limits (European Food Safety Authority (EFSA), 2008; Matthäus and Angelini, 2005). On the other hand, the glucosinolate content of the seeds, similar to other specialized metabolites of camelina, show a very high plasticity due to environmental conditions and in different genotypes (Boutet *et al*., 2022). Therefore, it is desirable to generate new camelina lines showing a stable and reduced glucosinolate content. In analogy, breeding programmes of rapeseed resulted in the generation of lines with low glucosinolate and erucic acid contents in the 1970s. The oil of these improved canola lines is low in erucic acid (<2%), the dried seeds contain <20 mmol/kg and the defatted seed meal <30 mmol/kg of glucosinolates (European Food Safety Authority (EFSA), 2008). The glucosinolate content in the feed especially for monogastric animals has been restricted to 1–1.5 mmol/kg and even less for young animals (European Food Safety Authority (EFSA), 2008).

The biological function of glucosinolates includes plant defence against herbivores and pathogens. Glucosinolates are sulphur‐ and nitrogen‐containing plant metabolites with a characteristic core structure and a variable side chain derived from one of eight amino acids. Depending on the precursor amino acid, they can be classified as aliphatic (methionine, alanine, valine, leucine, isoleucine), aromatic (tyrosine, phenylalanine) or indole‐derived (tryptophan) glucosinolates (Halkier and Gershenzon, 2006). In Arabidopsis, glucosinolates are found in all plant organs during vegetative development and during seed production (Brown *et al*., 2003). Because Arabidopsis seeds cannot synthesize glucosinolates, they are imported via two glucosinolate transporters, AtGTR1 and AtGTR2, which are closely related, high affinity proton/glucosinolate symporters (Nour‐Eldin *et al*., 2006, 2012; Nour‐Eldin and Halkier, 2009). Double mutants of *atgtr1atgtr2* lack glucosinolates in their seeds accompanied with a 10‐fold accumulation of methionine‐derived (aliphatic) glucosinolates in the leaves and silique walls. The two transporters are plasma membrane‐localized and expressed in vascular tissues, while AtGTR1 is additionally expressed in adjacent mesophyll cells. Glucosinolates are transported in the phloem and xylem over long distances from leaves to seeds and between leaves and roots (Andersen *et al*., 2013; Chen *et al*., 2001; Nour‐Eldin *et al*., 2012). The biosynthesis of aliphatic glucosinolates is initiated by one or several rounds of chain elongation reactions that start with the transamination of the amino acid yielding the corresponding 2‐ketoacid. The 2‐ketoacid undergoes condensation with acetyl‐CoA, catalysed by methylthioalkylmalate synthase 1 (MAM1; Grubb and Abel, 2006; Nour‐Eldin and Halkier, 2009). Isomerization, oxidative decarboxylation and another transamination reaction result in the homologous amino acid elongated by one CH_2_ group. In a second set of reactions including oxidation/decarboxylation, oxidation/conjugation, C‐S cleavage, glucosylation and sulfatation, the core structure of glucosinolates, R‐CH(‐S‐glucosyl) = N‐${\mathrm{OSO}}_{3}^{-}$, is formed. Finally, the side chain of the glucosinolates can be modified in further reactions. Glucosinolate biosynthesis is regulated by R2R3‐type MYB transcription factors, in particular by six members of the subgroup 12 (Stracke *et al*., 2001). AtMYB28, AtMYB29 and AtMYB76 are positive regulators of the biosynthesis of aliphatic glucosinolates in Arabidopsis, and AtMYB34, AtMYB51 and AtMYB122 are involved in the biosynthesis of indole glucosinolates (Frerigmann and Gigolashvili, 2014; Gigolashvili *et al*., 2007, 2008). AtMYB28 plays the main role during the accumulation of long‐chain aliphatic glucosinolates. As a consequence, long‐chain aliphatic glucosinolates are absent from the *atmyb28* mutant, while the amounts of short‐chain aliphatic glucosinolates are partially reduced. On the other hand, only short‐chain aliphatic glucosinolates are affected in the *atmyb29* mutant (Beekwilder *et al*., 2008; Sønderby *et al*., 2007). In contrast to the single mutants, *atmyb28atmyb29* double mutants lack all aliphatic glucosinolates. AtMYB76 plays only a minor role in the regulation of the synthesis of aliphatic glucosinolates in Arabidopsis (Gigolashvili *et al*., 2008).

To reduce the glucosinolate levels in camelina seeds, we embarked on a Cas9‐mediated mutagenesis approach by pursuing two strategies: (i) the reduction in glucosinolate transport into the seeds by targeting the transporter genes *GTR1*, *GTR2*; and (ii) the down‐regulation of glucosinolate biosynthesis by targeted mutagenesis of the *MYB28* and *MYB29* transcription factor genes. In contrast to *Arabidopsis*, camelina harbours a hexaploid genome, which increases the complexity of the Cas9‐based gene disruption approach. After transformation of camelina plants with the *cas9*/gRNA constructs, T‐DNA‐free homozygous or heteroallelic knockout mutants were obtained via segregation. Analyses of seeds and roots revealed that the mutant lines contained strongly and differentially reduced contents of glucosinolates in the seeds, in some lines with a decrease down to zero, without affecting the oil and protein contents of the seeds.

## Results

### Expression patterns of the homeologs of glucosinolate transporters GTR1, GTR2 and the transcription factor MYB28 of glucosinolate biosynthesis

The two glucosinolate transporters AtGTR1 and AtGTR2 are involved in the translocation and accumulation of glucosinolates in Arabidopsis seeds. As a consequence, the Arabidopsis *atgtr1atgtr2* seeds lack glucosinolates (Nour‐Eldin *et al*., 2012). The transcription factors AtMYB28 and AtMYB29 are positive regulators of the biosynthesis of aliphatic glucosinolates in Arabidopsis (Gigolashvili *et al*., 2007; Hirai *et al*., 2007). Seeds of *atmyb28 atmyb29* lack aliphatic glucosinolates. Protein BLAST searches were performed against the *Camelina sativa* genome sequence in the Genbank database using the orthologous sequences of *AtGTR1*, *AtGTR2*, *AtMYB28* and *AtMYB29* from Arabidopsis. Because of the hexaploid structure of its genome, camelina is expected to harbour three homeologous loci corresponding to each orthologous gene from Arabidopsis (Kagale *et al*., 2014). Consequently, three homeologs were identified in camelina each for *GTR1* (*CsGTR1‐A*, *CsGTR1‐B*, *CsGTR1‐C*), *GTR2* (*CsGTR2‐A*, *CsGTR2‐B*, *CsGTR2‐C*), *MYB28* (*CsMYB28‐A*, *CsMYB28‐B*, *CsMYB28‐C*) and MYB29 (*CsMYB29‐A*, *CsMYB29‐B*, *CsMYB29‐C*).

**Figure 1:**
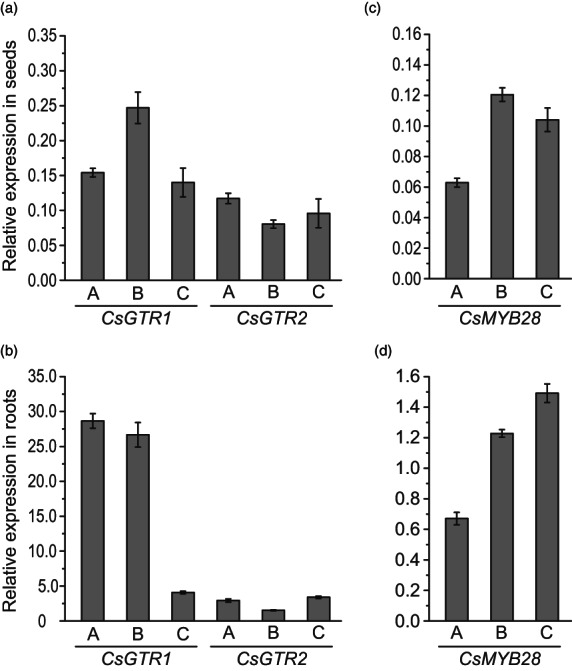
Relative expression of *CsGTR1*, *CsGTR2* and *CsMYB28* in seeds and roots of camelina wild type. Relative expression in seeds and roots of the three homeologs (A, B, C) of *CsGTR1* and *CsGTR2* (a, b) and of the three homeologs (A, B, C) of *CsMYB28* (c, d) was determined using the ΔΔ*c*_t_ value relative to α‐tubulin. Mean and SD, *n* = 5. Green seeds were harvested in the mature embryo stage and roots were used from adult plants.

To study the expression of the *CsGTR1*, *CsGTR2* and *CsMYB28* homeologs in different tissues, RNA was extracted from green seeds and roots of wild type (WT), and expression levels were determined by quantitative real‐time PCR (qPCR) using homeolog‐specific primers. All homeologs were more highly expressed in roots compared with the moderate expression in the seeds (Figure 1). The expression of *CsGTR1‐C* und *CsGTR2* (*A*, *B*, *C*) in the roots was about 10‐fold higher, and more than 100‐fold higher for *CsGTR1‐A* and *B*. Therefore, *CsGTR1‐A* and *CsGTR1‐B* represent the predominating activities in the roots. The expression of the three homeologous *CsMYB28* genes in the seeds was also lower by a factor of ~10 compared with roots.

### Cas9‐mediated mutagenesis of *CsGTR1* , *CsGTR2* , *CsMYB28* and *CsMYB29* homeologs in camelina

**Figure 2:**
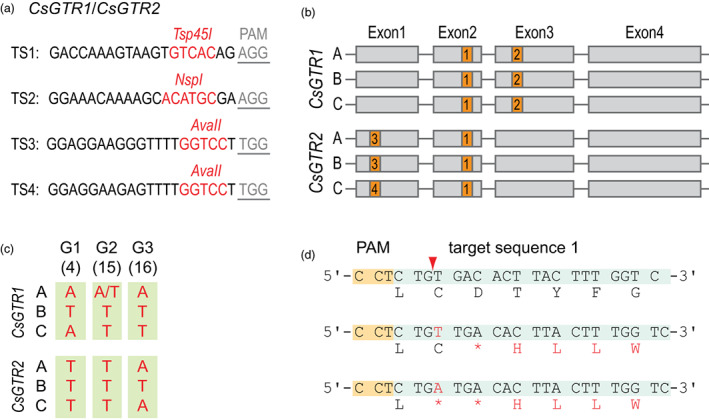
Cas9‐mediated site‐directed mutagenesis of *CsGTR1/CsGTR2* in camelina. (a) Target sequences (TS1‐4) in the three homeologs each of *CsGTR1* and *CsGTR2* were selected which contain recognition sites of restriction enzymes (red). The protospacer‐adjacent motifs (PAM) are underlined. (b) The orange boxes highlight the localizations of the TS1‐4 in the exons of *CsGTR1* and *CsGTR2*. (c) Two types of mutations (T or A insertions) were found in the three homeologs (A, B, C) of *CsGTR1* and *CsGTR2* in the T2 lines of G1, G2 and G3. A/T indicates a biallelic mutation. (d) Insertions of single A in the TS1 cause a premature stop codon after amino acid 135 (CsGTR1‐A) or 137 (CsGTR1‐B, C) or 105 (CsGTR2‐A, B, C) and insertion of a T results in a stop codon after amino acids 136 (CsGTR2‐A), 138 (CsGTR2‐B, C) or 106 (CsGTR2‐A, B, C). The red arrowhead indicates the position of the Cas9 cleavage site in the TS1. The mutations and altered amino acids are shown in red, premature stops codons are indicated with an asterisk.

**Figure 3:**
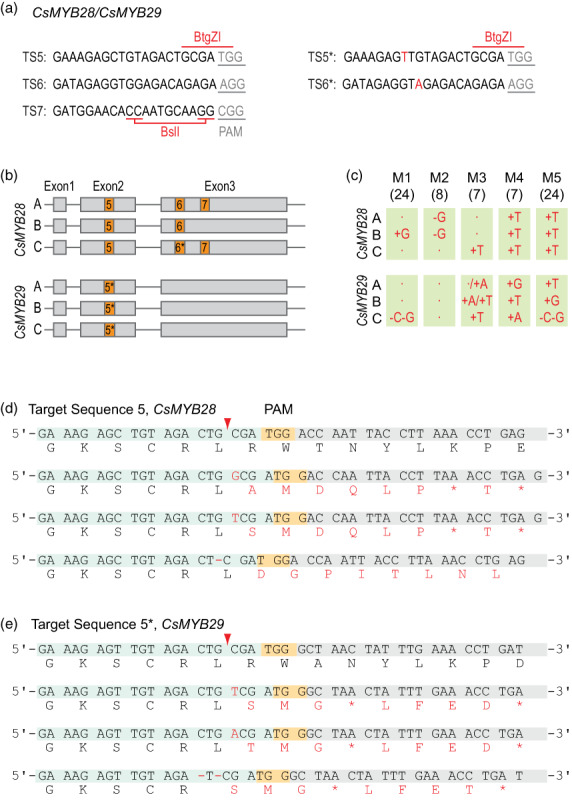
Cas9‐mediated site‐directed mutagenesis of *CsMYB28/CsMYB29* in camelina. (a) Target sequences TS5‐7 present in the three homeologs each of *CsMYB28* and TS5* in *CsMYB29*. (b) Localization of TS5‐7 (orange boxes) in the exons of *CsMYB28A‐C*, and of TS5* (with 1 bp mismatch to TS5) in exon 2 of *CsMYB29A‐C*. (c) Different insertions (+base) or deletions (−base) were found in TS5 and TS5* of the *CsMYB28* or *CsMYB29* homeologs (A, B, C) in the T3 plants M1‐M5, respectively. The numbers in brackets denote the T1 progenitor lines. Comprehensive sequence information for mutations in TS5, TS5*, TS6 and TS6* is shown in Figure S5. (d) Single‐base insertions (T, G) or deletions (G) in TS5 present in all *CsMYB28* homeologs lead to altered protein sequences downstream of amino acid L55 and premature stop codons. (e) Single‐base insertions (T, A, G) or deletions (G, C) in TS5* of the *CsMYB29* homeologs similarly cause amino acid mutations and premature stop codons after amino acid L55. The red arrowheads indicate the positions of the Cas9 cleavage sites in the TS5 and TS5*.

Four target sequences (TSs) were selected to mutagenize the homeologs of *CsGTR1* and *CsGTR2*, and three TSs for the mutagenesis of *CsMYB28* and *CsMYB29* (Figures 2 and 3; Sequences S1). All TSs are located within the open reading frames (Figures 2 and 3), and most of the TSs contain recognition sites for restriction enzymes close to the protospacer adjacent motif (PAM) to aid in the screening for mutations. The target‐specified guide RNA (gRNA) sequences were cloned into a binary vector harbouring the *cas9* gene under control of a ubiquitin promoter from parsley (Figure S1; Kawalleck *et al*., 1993). The binary vectors targeting *CsGTR1* and *CsGTR2* harboured four gRNAs, while the vector for *CsMYB28* and *CsMYB29* contained three gRNAs. *Agrobacterium*‐mediated transformation of camelina with the binary vectors for mutagenesis of *CsGTR1/CsGTR2* and *CsMYB28/CsMYB29* resulted in 17 and 26 independent T1 plants, respectively. Genomic DNA from leaves of the transgenic plants was extracted to amplify the TS‐containing regions of *CsGTR1‐A*, *CsGTR2‐C* or *CsMYB28‐A* (Table S1). The PCR products of *CsGTR1‐A* and *CsGTR2‐C* were digested with *Tsp45*I, and *CsMYB28‐A* PCR products with *BtgZ*I. The recognition sites of the two enzymes are located in TS1 or TS5, respectively (Figures 2 and 3).

### Isolation of *csgtr1csgtr2* mutant lines of camelina

Screening of T1 plants after transformation with the CsGTR1/CsGTR2 construct resulted in the identification of five lines with mutations in the *Tsp45*I restriction sites of *CsGTR1‐A*, and eight lines with mutations in *CsGTR2‐C* (Figure S2a,b). Four of these lines (lines 4, 13, 15 and 16) showed mutations in both genes, *CsGTR1* and *CsGTR2*. T2 plants were grown from seeds of lines 4, 13, 15 and 16 to obtain homozygous, homoallelic or heteroallelic mutant lines of the six *CsGTR1* and *CsGTR2* homeologs. T2 lines were screened by digestion and sequencing of the PCR products for all six homeologs of *CsGTR1* and *CsGTR2*. Nine of the T2 plants contained homozygous or biallelic mutations in all six homeologs of *CsGTR1* and *CsGTR2*. Five of the plants were grown from non‐transgenic seeds and were thus free of transgenes. An example (line 16) of the restriction analyses is shown in Figure S2c. The most frequent mutations in TS1 (shared by all homeologs of *GTR1* and *GTR2*) were single‐bp insertions of T (~60%) or A (~20%). Besides, a few events featured other insertions (+C or +27 bps) or short deletions (−GACAC, −TG, −19 bp). No mutations were found in TS2, TS3 or TS4 in these plants suggesting a low efficiency of the corresponding guide RNAs. Based on these results, three independent T‐DNA‐free lines designated G1, G2 and G3, which are descendants of lines 4, 15 and 16, respectively, were selected. The mutations in these lines are homozygous, and only the locus *CsGTR1‐A* in line G2 was biallelic with A or T insertions (Figures 2c and S3a). The mutations in all selected T‐DNA‐free lines were obtained by inheritance, indicating that the mutations were already present in the reproductive tissues of the T1 generation.

Frameshift mutations generally entail downstream non‐sense amino acid sequences. In addition, single A insertions in TS1 result in two premature stop codons after amino acids 135 (CsGTR1‐A) or 137 (CsGTR1‐B, C), single T insertions cause one stop codon after amino acids 136 (CsGTR1‐A) or 138 (CsGTR1‐B, C; Figure 2d). The intact CsGTR1‐A protein consists of 646, and CsGTR1‐B and CsGTR1‐C each of 648 amino acids. CsGTR2 (A, B, C) proteins comprise 614, and their truncated versions 105 or 106 amino acids depending on A or T insertions, respectively. The truncated CsGTR1 and CsGTR2 proteins still contain the ETFEK motif which is essential for proton coupling and active glucosinolate transport (Jørgensen *et al*., 2015). However, further amino acids required for transport and protein stability are eliminated and transmembrane domains abolished (Chung *et al*., 2021). Therefore, the mutant loci presumably represent null alleles.

### Isolation of *csmyb28csmyb29* mutant plants of camelina

Similar as described for the mutagenesis for *CsGTR1* and *CsGTR2*, six independent T1 lines with mutations in *CsMYB28‐A* were identified after screening by sequencing and *BtgZ*I digestion of PCR products of TS5. For three lines (7, 8, 24), T2 offspring seeds were germinated to obtain plants with homozygous or heteroallelic mutations in one, two or all three *CsMYB28* homeologs. Five T‐DNA‐free T3 mutant plants (designated M1–M5) were selected. The plants M1 (line 24) and M3 (7) are single mutants of *CsMYB28‐B* and *C*, respectively, M2 (8) is a double mutant of *CsMYB28‐A* and *B*, and M4 (7) and M5 (24) represent triple mutants in all three homeologs (Figures 3 and S4). For TS5, we found +T insertions (~70%), and single events of an insertion (+G, +C) or a deletion (−G) and WT alleles. *CsMYB28‐C* does not contain a *bona fide* TS6, instead a sequence motif is present in this locus with 1 bp difference to TS6 (designated TS6*). Mutations in TS6/TS6* were mostly single nucleotide insertions (~33% +C insertions; ~20% −CT deletions; ~15% +T insertions; ~10% +A insertions), the remaining lines carried WT alleles. For TS7, no mutation was found in these plants, indicating low mutagenesis efficiency. Next, the mutations in TS5*, which differs to TS5 by one mismatch, were analysed for the three loci of CsMYB29 in the plants M1–M5 (Figures 3 and S4). The plant M1 carries two single deletions in CsMYB29‐C, plant M2 is devoid of any mutations in CsMYB29‐A, B or C. The plant M3 harbours a heterozygous insertion in locus CsMYB29‐A, and homozygous insertions in the B and C homeologs. The other two plants (M4, M5) carry insertions/deletions in all three homeologs of CsMYB29. The TS5* mutations mostly reveal single base insertions (T, A, G) or simultaneous deletions of two separate bases (−C, −G).

The insertions or deletions lead to non‐sense protein sequences after amino acid 55 in the CsMYB28 and CsMYB29 homeologs including premature stop codons (Figure 3). The three homoelogous WT proteins of CsMYB28 comprise each 370 amino acids, while the CsMYB28‐A, B and C homeologs harbour 341, 342 and 340 amino acids, respectively. All these mutations lead to the disruption of the conserved domain structure characteristic for R2R3‐type MYB transcription factors and consequently to protein inactivation (Stracke *et al*., 2001). Further mutations that simultaneously occurred in TS6 or TS6* in the *CsMYB28* homeologs do not restore the original protein sequences altered by mutations in TS5.

### Decreased glucosinolate contents in seeds and roots of *csgtr1csgtr2* mutants

**Figure 4:**
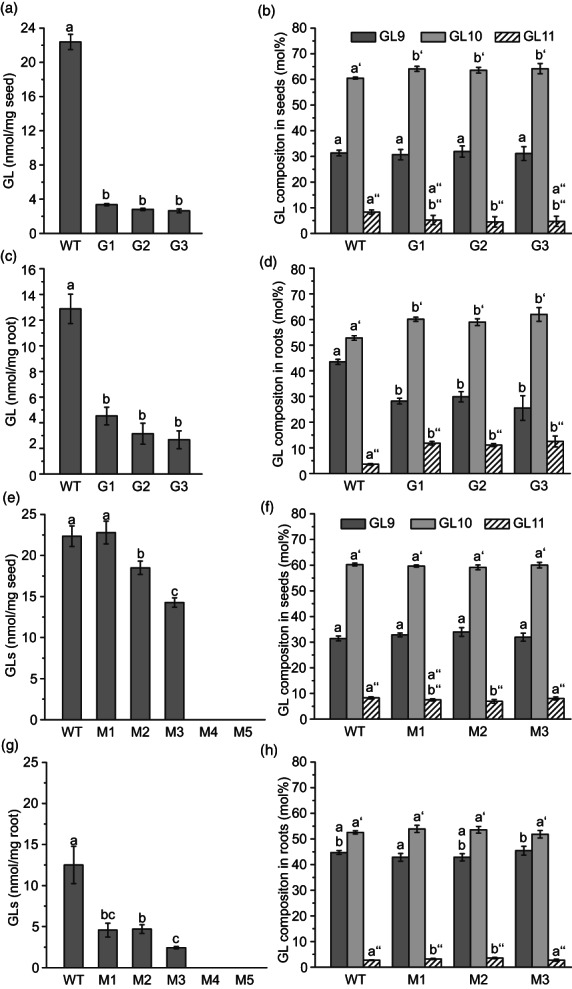
Glucosinolate (GL) contents of seeds and roots from *csgtr1csgtr2* and *csmyb28csmyb29* mutant lines. (a) Glucosinolate content of *csgtr1csgtr2* lines per seed dry weight (*n* = 4; *P* < 0.01). (b) Seed glucosinolate composition of *csgtr1csgtr2* lines (*n* = 4; *P* < 0.05). (c) Glucosinolate content per root dry weight of *csgtr1csgtr2* lines (*n* = 5; *P* < 0.01). (d) Root glucosinolate composition of *csgtr1csgtr2* lines (*n* = 5; *P* < 0.01; ANOVA and post hoc Tukey test). (e) Glucosinolate content of *csmyb28csmyb29* lines per seed dry weight (*n* = 5; *P* < 0.01). (f) Seed glucosinolate composition of *csmyb28csmyb29* lines (*n* = 5; *P* < 0.05). (g) Glucosinolate content per root dry weight (*n* = 5; *P* < 0.05). (h) Root glucosinolate composition of *csmyb28csmyb29* lines (*n* = 5; *P* < 0.05). ANOVA and post hoc Tukey test. Different letters indicate significant differences. Note that seeds and roots of *csmyb28csmyb29* lines M4 and M5 completely lack glucosinolates. All measurements were performed with dried T4 seeds or with roots from T3 plants of *csgtr1csgtr2* lines or with T5 seeds or roots sampled from T4 plants of *csmyb28csmyb29* lines.

Glucosinolates were measured in different plant tissues of *csgtr1csgtr2* mutants via HPLC after purification and desulfatation. The amounts of total glucosinolates were reduced to 3.4, 2.8 and 2.6 nmol/mg seeds in the G1, G2 and G3 lines, respectively, while WT seeds contained 22.4 nmol/mg, corresponding to a reduction by 85%–88% (Figure 4a). Therefore, small amounts of glucosinolates were detected in the seeds of the camelina lines G1, G2 and G3, in contrast to the seeds of the Arabidopsis double mutant *atgtr1atgtr2* which completely lacked glucosinolates (Nour‐Eldin *et al*., 2012). Because all three homeologs of CsGTR1 and CsGTR2 in the mutant lines carry null mutations, the accumulation of low levels of glucosinolate cannot be explained by residual activities of CsGTR1 or CsGTR2. The glucosinolate distribution in the mutant seeds was hardly affected, with only slight increases in GL10 and slight decreases in GL11 compared with the WT that contained 31.3, 60.5 and 8.3 nmol/mg of GL9, GL10 and GL11, respectively (Figure 4b).

Next, glucosinolates were measured in leaves, stems and roots of the *csgtr1csgtr2* lines. While glucosinolates were undetectable in leaves and stems of camelina WT, we found notable amounts of glucosinolates in the roots of WT [12.9 nmol/mg dry weight (DW); Figure 3c]. This amount represents more than half of the glucosinolate levels compared with seeds (22.4 nmol/mg) and is in accordance with previous reports (Czerniawski *et al*., 2021). The roots of the three lines G1, G2 and G3 revealed glucosinolate contents of 4.5, 3.1 and 2.7 nmol/mg DW, respectively, corresponding to 35.2%, 24.2% and 20.8% of the levels in WT. The finding that the relative decrease in glucosinolates in roots was less pronounced than in seeds indicates that root glucosinolates are less severely affected by the *csgtr1csgtr2* mutations. Similar to the seeds, roots contained the aliphatic glucosinolates GL9, GL10 and GL11, but with a different composition of 43.5, 52.8 and 3.6 mol% of total glucosinolates in WT roots, respectively, compared with 31.3, 60.5 and 8.3 mol% in seeds. Therefore, the level of GL9 is elevated in roots at the expense of GL10 and GL11 (Figure 4d). Interestingly, the glucosinolate composition in the *csgtr1csgtr2* roots was altered compared with WT, because the amount GL9 was strongly reduced while the levels of GL10 and GL11 were elevated. The proportions of GL9, GL10 and GL11 in the *csgtr1csgtr2* roots from line G2, were 29.9, 59.0 and 11.1 mol%, respectively, thus resembling the composition in WT seeds.

Taken together, the mutations in CsGTR1 and CsGTR2 led to a strong reduction in glucosinolates by 85%–88% in mutant seeds without affecting the distribution of GL9, GL10 and GL11. Considerable amounts of GL9, GL10 and GL11 were detected in the roots of camelina WT, however, with a different composition compared with seeds. The levels of glucosinolates in mutant roots were also strongly reduced, accompanied by a change in their composition.

### The *csgtr1csgtr2* mutant embryos accumulate low amounts of glucosinolates

**Figure 5:**
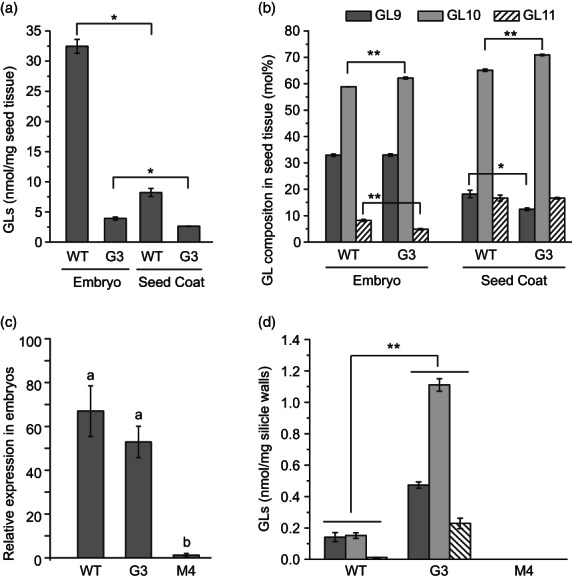
Glucosinolate content and expression of glucosinolate biosynthetic genes in seeds and silicles. (a) Glucosinolate content per weight of seed tissue of *csgtr1csgtr2* line G3; (*n* = 3; *P* < 0.001; Student's *t*‐test). (b) Glucosinolate composition of embryo and seed coat of *csgtr1csgtr2* line G3. Values from wild‐type (WT) and mutant embryos, and values from WT and mutant seed coats were compared (**: *P* < 0.01; *: *P* < 0.05; Student's *t*‐test). (c) Relative expression of the glucosinolate biosynthetic gene *CsMAM1* in green embryos of camelina WT and mutants. *CsMAM1* expression in green embryos of WT and *csgtr1csgtr2* mutant line G3 is increased relative to the *csmyb28* null mutant line M4. The green embryos (T4) were harvested and pooled per line. ANOVA and post hoc Tukey test. Letters indicate significant differences (*n* = 4; *P* < 0.01). (d) Increased contents of the glucosinolates GL9, GL10, GL11 in silicle walls (carpels/valves) of *csgtr1csgtr2* line G3 (total, 1.81 nmol/mg DW) compared to WT (0.30 nmol/mg DW) (*n* = 4; *P* < 0.001; Student's *t*‐test). GL9, GL10 and GL11 were not detectable in silicle walls of the *csmyb28csmyb29* line M4.

Seeds of the Arabidopsis double mutant *atgtr1atgtr2* completely lack glucosinolates, because of a block in the translocation from maternal tissues to the seeds (Nour‐Eldin *et al*., 2012). As a consequence, source tissues like leaves or silique walls of *atgtr1atgtr2* plants accumulate more than 10 fold of the amounts of glucosinolates compared with WT. The detection of low amounts of glucosinolates in camelina *csgtr1csgtr2* seeds (Figure 4) led to the question, whether these glucosinolates might be restricted to maternal tissues (seed coat) or the embryo. Therefore, seeds from camelina WT and mutant line G3 were dissected into embryos and seed coats, and the glucosinolates were measured. We detected low amounts of glucosinolates in the mutant seed coats with 2.6 nmol/mg DW (31.7% of WT; 8.2 nmol/mg DW) and in the mutant embryos with 3.9 nmol/mg DW (12.0% of WT embryos; 32.5 nmol/mg DW; Figure 5a). Therefore, the reduction in glucosinolates is less pronounced in the mutant seed coats (by 68.3%) than in the mutant embryos (by 88%, compared with WT). Besides, differences in the glucosinolate composition were observed between embryos and seed coats, independent from the genetic background. The seed coats of WT showed a >40% lower content of GL9, slightly elevated levels of GL10, and a 50% increase in GL11 compared with whole seeds, whereas the embryos resemble whole seeds in their glucosinolate composition (Figure 5b). Therefore, glucosinolates are found both in embryos and seed coats (maternal tissue) of WT and in lower amounts of *csgtr1csgtr2* mutants. Besides, the *csgtr1csgtr2* mutant embryos are more affected by the reduction in glucosinolates than the mutant seed coats.

To address the question whether camelina embryos can synthesize glucosinolates, the expression of *METHYLTHIOALKYLMALATE SYNTHASE 1* (*MAM1*) involved in aliphatic glucosinolate biosynthesis (Nour‐Eldin and Halkier, 2009) was studied by qPCR using primer sequences conserved in the three *CsMAM1* homeologs. The *CsMAM1* expression in embryos of WT and line G3 was similar, whereas it was ~60‐fold lower in the *csmyb28csmyb29* null mutant M4. (Figure 5c). The reduced expression of *CsMAM1* in *csmyb28csmyb29* is in line with the result that *AtMAM1* expression depends on AtMYB28 in Arabidopsis (Gigolashvili *et al*., 2007). The expression of *CsMAM1* in WT and G3 indicates that the embryos can synthesize glucosinolates, contributing to glucosinolate accumulation in the seeds, similar to seeds of *Sinapis alba* (Du and Halkier, 1998). The complete loss of glucosinolates in *atgtr1atgtr2* seeds and the concomitant increase in leaves and silique walls has been explained by a block in the long‐distance transport of glucosinolates (Chen *et al*., 2001; Gigolashvili *et al*., 2007; Nour‐Eldin *et al*., 2012; Nour‐Eldin and Halkier, 2009). Glucosinolates were not detectable in leaves or stems of camelina WT and *csgtr1csgtr2* mutants. However, a considerable increase of glucosinolates in the silicle walls of line G3 (1.81 nmol/mg; WT, 0.30 nmol/mg; Figure 5d) was detected. Therefore, silicle walls of camelina presumably act as source tissue of glucosinolates transported to the seeds by CsGTR1 and CsGTR2.

### Elimination of glucosinolates from *csmyb28csmyb29* mutant plants

Next, glucosinolates were measured in the seeds and roots of the camelina *csmyb28csmyb29* mutant plants (Figure 4e,d). We observed a complete loss of glucosinolates in the seeds and the roots of the *csmyb28csmyb29* mutant lines M4 and M5 which are disrupted in all three homeologs of *CsMYB28* and *CsMYB29* each (in the following referred to as hextuple mutants). This result is in line with the Arabidopsis mutant *atmyb28atmyb29* which shows a complete loss of aliphatic glucosinolates (Sønderby *et al*., 2007). A partial reduction in glucosinolates was observed in the other three *csmyb28csmyb29* mutants of camelina (M1, M2, M3) carrying mutations in one or more *CsMYB28* and *CsMYB29* homeologs. Close inspection of the glucosinolate contents in the individual lines revealed that the *CsMYB28* and *CsMYB29* homeologs contribute to different extents to the accumulation of glucosinolates. While the glucosinolate content in seeds of the mutant M1 disrupted in *CsMYB28‐B* and *CsMYB29‐C* was not affected, line M3, which is mutagenized in *CsMYB28‐C*, *CsMYB29‐A* (heterozygous), *B* and *C* (both homozygous), showed a decrease in glucosinolate levels to 14.3 nmol/mg (63.9% of WT; 22.3 nmol/mg). In contrast, line M2, which is disrupted in the two homeologs *CsMYB28‐A* and *B*, was less severely affected with a glucosinolate content of 18.5 nmol/mg (82.8% of WT). In the roots, more pronounced decreases in the glucosinolate levels were observed. The roots of lines M1, M2 and M3 contained 4.6, 4.7 and 2.4 nmol/mg glucosinolates, respectively, corresponding to 36.6%, 37.5% and 19.4% of WT level (12.5 nmol/mg). The glucosinolate composition in seeds and roots of all lines was very similar to WT (Figure 4).

Altogether, these experiments show that the deletion of the six *CsMYB28* and *CsMYB29* homeologs leads to a complete loss of glucosinolates in seeds and roots. Single and multiple mutants of *csmyb28csmyb29* showed a moderate reduction in glucosinolates in seeds and a more severe reduction in the roots. The mutagenesis of the *CsMYB28* and *CsMYB29* homeologs does not affect the composition of glucosinolates in seeds and roots.

### Seed oil and protein contents are not affected in *csgtr1csgtr2* and *csmyb28csmyb29* mutant lines of camelina

**Figure 6:**
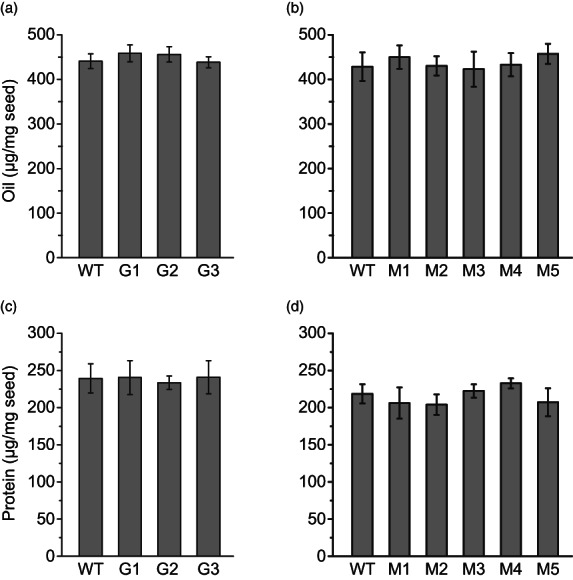
Oil and protein contents of *csgtr1csgtr2* (G1, G2, G3) and *csmyb28csmyb29* (M1–M5) mutant lines. (a) and (b) Oil content per seed weight. Fatty acid methyl esters were measured by GC. (c) and (d) Protein content per seed weight. Protein amounts were determined using Lowry assay. T4 seeds from *csgtr1csgtr2* and T5 seeds from *csmyb28csmyb29* mutant plants were used, and three seeds were pooled per measurement. The values are not significantly different (ANOVA; post hoc Tukey; *n* = 5). The slight differences in the oil and protein contents of corresponding mutants are negatively correlated (correlation coefficient = −0.53).

It is of high importance that the gain in quality via mutagenesis of *CsGTR1*, *CsGTR2*, *CsMYB28* or *CsMYB29* does not entail a yield penalty in camelina seeds. Therefore, oil and protein contents were measured in the seeds. The oil contents in *csgtr1csgtr2* and *csmyb28csmyb29* seeds ranged from 439–459 and 423–457 nmol/mg, respectively, compared to 429–441 nmol/mg in WT seeds (Figure 6a,b). The fatty acid profiles of *csgtr1csgtr2* and *csmyb28csmyb29* mutant seeds were unchanged in comparison with WT (Figure S5). Furthermore, the *csgtr1csgtr2* and *csmyb28csmyb29* mutant seeds contained 234–241 and 204–233 nmol/mg of protein, respectively, compared with 219–239 nmol/mg for WT seeds (Figure 6c,d). Therefore, seed oil and protein contents were not affected in *csgtr1csgtr2* and *csmyb28csmyb29* mutant lines of camelina.

## Discussion

Because rapeseed and other *Brassica* species lack long‐chain aliphatic glucosinolates, data on the toxicity of these specialized metabolites as found in camelina are limited. Hydrolysis of long‐chain glucosinolates leads to the production of non‐volatile isothiocyanates and nitriles which were identified in camelina seed extracts and considered to be toxic (Amyot *et al*., 2019; Matthäus and Zubr, 2000). Especially GL10 hydrolysis products have cytotoxic effects on mouse hepatoma cells in a concentration of 5 μm (Das *et al*., 2014). Besides, impaired growth was observed for broiler chickens when fed with 5% of camelina seed cake (Ryhänen *et al*., 2007). Camelina seeds exhibit a higher glucosinolate content (13.2–36.2 mmol/kg seed meal, depending on the variety) than canola (7.2 mmol/kg; Boutet *et al*., 2022; Colombini *et al*., 2014; Schuster and Friedt, 1998). We obtained comparable values for seeds of camelina WT139 (22.4 nmol/mg). Mutagenesis of the three homeologs of each of the two glucosinolate transporter genes in the camelina *csgtr1csgtr2* mutants resulted in a considerable reduction in glucosinolates to levels of 2.6–3.4 nmol/mg seeds. A complete elimination of glucosinolates was achieved in *csmyb28csmyb29* mutant seeds. Thus, this is the first report on glucosinolate‐free seeds from crop plants of the Brassicaceae family. These results represent a considerable improvement of the quality of camelina seed products which meet or even exceed the international standards for glucosinolate contents (European Food Safety Authority (EFSA), 2008). Camelina seeds are low in other antinutritional compounds such as phytic acid, condensed tannins, sinapine and erucic acid (Colombini *et al*., 2014; Russo and Reggiani, 2012; Vollmann and Eynck, 2015). All these favourable traits in combination with the reduced glucosinolate content certify that the seeds of the mutant plants meet canola quality. The mutant lines of the present study were transgene‐free as deduced from the absence of the DsRed marker after segregation. Therefore, introduction of the Cas9‐derived mutant lines into the field is feasible according to legislation in many countries (Menz *et al*., 2020; Ruffell, 2018).

In contrast to the seeds of the Arabidopsis double mutant *atgtr1atgtr2* that lacks glucosinolates, seeds of the camelina *csgtr1csgtr2* mutants are still accumulating reduced amounts of glucosinolates (Figure 4). The loss of glucosinolates in *atgtr1atgtr2* seeds and the concomitant increase in leaves and silique walls was explained by a block in the long‐distance transport of glucosinolates from maternal tissues to the seeds (Chen *et al*., 2001; Nour‐Eldin *et al*., 2012). Long‐distance transport from leaves or roots can be excluded for camelina, because neither the *csgtr1csgtr2* mutant plants nor WT accumulate glucosinolates in their leaves or roots (Andersen *et al*., 2013). The less pronounced reduction in glucosinolates in the mutant seed coat (by 68.2%) compared to the embryo (by 88%), suggests that glucosinolates are retained in the mutant seed coat due to a block in the transport by CsGTR1 and CsGTR2 (Figure 5). The silicle walls of the *csgtr1csgtr2* line G3 showed an up to six‐fold accumulation of glucosinolates compared with WT (Figure 5d) indicating that silicle walls are a further source of seed glucosinolates in camelina similar to Arabidopsis (Nour‐Eldin and Halkier, 2009).

The presence of glucosinolates in the embryo may be derived from alternative import mechanisms. In fact, diffusion between maternal (integuments) tissue and the embryo has previously been ruled out (Radchuk and Borisjuk, 2014). On the other hand, alternative transporters might be involved in transferring glucosinolates into the seeds. Camelina contains three homeologs with sequence similarities to an additional glucosinolate transporter (AtGTR3). AtGTR3 reveals specificity towards indole glucosinolates (Jørgensen *et al*., 2017) which were recently found in inflorescences of camelina (Czerniawski *et al*., 2021). However, the involvement of putative indole‐glucosinolate specific AtGTR3‐related transporters in the accumulation of aliphatic glucosinolates in camelina seeds remains elusive. The initial steps of glucosinolate biosynthesis take place in maternal tissues of Arabidopsis, whereas the seeds need to import glucosinolates and can only modify the glucosinolate structure (Nour‐Eldin and Halkier, 2009). In some species, for example *Sinapis alba*, the seeds are able to synthesize low amounts of glucosinolates (Du and Halkier, 1998). The detection of considerable levels of *CsMAM1* transcripts in the embryos of WT and *csgtr1csgtr2* mutants (Figure 4c) indicates that camelina embryos are capable of performing glucosinolate biosynthesis. Thus, these findings suggest that two pathways (i.e. the transport by CsGTR1 and CsGTR2 and the *de novo* synthesis) contribute to the accumulation of glucosinolates in the seeds of camelina.

The transcription factors AtMYB28, AtMYB29 and AtMYB76 are positive regulators of aliphatic glucosinolate synthesis in Arabidopsis, with overlapping functions but with distinct roles and lack of capability of complementing each other for the loss of glucosinolates in the respective mutants (Beekwilder *et al*., 2008; Gigolashvili *et al*., 2008; Sønderby *et al*., 2010). The disruption of all three homeologs of *CsMYB28* and *CsMYB29* each (in mutants M4 and M5) resulted in the complete loss of aliphatic glucosinolates. The various *CsMYB28* and *CsMYB29* homeologs presumably contribute to different extents to the synthesis of glucosinolates in camelina roots and seeds. The plant M2 only carries mutations in *CsMYB28‐A* and *B* and shows a minor reduction in glucosinolates in seeds, while root glucosinolate content is decreased to <40%. Therefore, *CsMYB28‐A* and *B* are highly relevant for glucosinolate production in the roots.

A recent study revealed that mutagenesis of two of the three homeologs of the *BoMYB28* gene in *Brassica oleracea* (broccoli) caused a decrease in glucosinolate contents. The leaves and florets of the broccoli plants carrying mutations in the *BoMYB28* homeologs C2 and C9 showed strong decreases in aliphatic glucosinolates, but indole derived‐glucosinolates were not significantly affected (Neequaye *et al*., 2021). The C7 homeolog of *BoMYB28* and the two homeologs of *BoMYB29* were not mutagenized in these plants. Therefore, the authors concluded that the *BoMYB28* homeologs are most relevant for the regulation of aliphatic glucosinolate biosynthesis in *Brassica oleracea*. The results obtained after mutagenesis of *BoMBY28* in broccoli are therefore in line with the data from Arabidopsis which showed that AtMYB28 is the most relevant factor for regulating aliphatic glucosinolate production, while AtMYB29 is specifically involved in short‐chain aliphatic glucosinolate synthesis (Beekwilder *et al*., 2008; Sønderby *et al*., 2010). The data presented here indicate that CsMYB28 exerts the predominant regulatory function for aliphatic glucosinolate biosynthesis at least in roots of camelina. In addition to seeds and roots, glucosinolates were recently found in young seedlings, inflorescences and silicles of camelina (Czerniawski *et al*., 2021). It is possible that CsMYB29 plays a role in regulating glucosinolate synthesis in these tissues. On the other hand, AtMYB76‐related sequences were not detected in the camelina genome by BLAST searches, and they were also not found in other *Brassica* species (Seo and Kim, 2017).

Glucosinolates protect plants from herbivores and pathogens. Therefore, breeding of low‐glucosinolate lines (as in the case of *Brassica* crops) have raised concerns about increased susceptibility to plant diseases (Augustine *et al*., 2013). The role of glucosinolates in the defence mechanism is complex (Hopkins *et al*., 2009). They can serve as repellents for generalists which prefer to feed on low glucosinolate lines, or they can function as attractants to crucifer specialists which are able to detoxify glucosinolates (Bodnaryk, 1997). Camelina is generally considered to be resistant to many diseases and pests. One reason is that camelina is not a preferred host for many insects because of the lack of glucosinolates in the areal vegetative tissues as stimulatory chemical cues. In addition, an antibiosis factor was suggested to be responsible for the low performance of different pests on camelina (Soroka *et al*., 2015). Moreover, two indole phytoalexins, camalexin and methoxy‐camalexin in camelina confer resistance to different diseases (Séguin‐Swartz *et al*., 2009). Camalexin is exclusively present in camelina and exhibits a higher toxicity than other phytoalexins. The two indole phytoalexins together with other antimicrobial compounds were also detected in the roots (Séguin‐Swartz *et al*., 2009). Therefore, despite the loss of root glucosinolates, the camelina *csgtr1csgtr2* and *csmyb28csmyb29* null mutants may still largely retain their resistance against pathogens.

## Experimental procedures

### Plant material and growth conditions

Seeds of *Camelina sativa* (accession CAM139; IPK Gatersleben, Germany) were sown on soil (Einheitserde ED73, Nitsch & Sohn, Kreuztal, Germany). Plants were grown in pots in the greenhouse under ambient light. During the winter period, additional light of 600 μmol s^−1^ m^−2^ for 16 h per day was applied.

### Plasmid construction for camelina transformation

The binary vector pHEE401E (Addgene plasmid # 71287, http://n2t.net/addgene:71287; RRID:Addgene_71 287) was obtained from Qi‐Jun Chen (China Agricultural University, Bejing, China). This vector contains an egg cell‐specific promoter for expression of the *cas9* endonuclease, an *AtU6‐26* promoter‐driven gRNA scaffold for insertion of target‐specific sequences, and a hygromycin B resistance cassette (Wang *et al*., 2015). The vector was modified by exchanging the egg cell promoter by a ubiquitin promoter and by the replacement of the hygromycin B resistance cassette with a DsRed marker. The ubiquitin promoter (PcUbi) from the Ubi4‐2 polyubquitin gene (Kawalleck *et al*., 1993) was amplified from genomic DNA of parsley (*Petroselinum crispum*) leaves with the primer pair bn3308/bn3309, introducing 5′‐*Spe*I and 3′‐*Kpn*I restriction sites (for oligonucleotides see Table S2). The exchange of the egg cell promoter (containing a 3′‐*Xba*I restriction site) by the PcUbi promoter (with 3′‐*Kpn*I) in pHEE401E required the re‐amplification of the *cas9* sequence with the primers bn3310/bn3311 (5′‐*Kpn*I/3′‐*Eco*RI). The PCR products of the *PcUbi* promoter and of *cas9* were sub‐cloned into pJet1.2 (Thermo Fisher Scientific, Dreieich, Germany), resulting in pJ‐PcUbi and pJ‐Cas9, respectively. The *PcUbi* sequence was released with *Kpn*I, *Bg*lI (internal site of pJet1.2) and inserted in pJ‐Cas9 linearized with *Kpn*I and *Bg*lI, resulting in pJ‐PcUbi‐Cas9. The cloning of the DsRed marker required the removal of a *Bsa*I restriction sequence from DsRed, because this site is also present in the cloning site for gRNA sequences of pHEE401E. Thus, the CaMV35S:DsRed sequence was amplified as two overlapping PCR products. Because of lacking suitable cloning sites in pHEE401E, additional amplification of sequences adjacent to the hygromycin B resistance cassette in pHEE401E was necessary. The 5′‐ and 3′‐adjacent sequences are intended to flank the 5′‐ and 3′‐ends (left and right flanking sequences) of the mutagenized CaMV35S:DsRed sequence, respectively, to allow the insertion into pHEE401E to replace the former hygromycin B resistance gene. These two adjacent sequences (named as *Eco*RI‐left‐pcr and right‐*Sac*II‐pcr, harbouring a 5′‐*Eco*RI and a 3′‐*Sac*II restriction site, respectively) were amplified as PCR products overlapping with CaMV35S:DsRed at its 5′‐ (left) and 3′‐ (right) ends with the primer pairs bn3312/bn3313 (*Eco*RI‐left‐pcr) and bn3318/bn3319 (right‐*Sac*II‐pcr), respectively, with pHEE401E as template. The primers bn3312 and bn3319 contain overhangs with *EcoR*I and a *Sac*II restriction sites, respectively. The two overlapping CaMV35S:DsRed sequences were amplified from the plant expression vector pBinGlyRed1‐IKU2 (Fatihi *et al*., 2013) with bn3314/bn3315 (DsRed‐mutA‐pcr) and bn3316/bn3317 (DsRed‐mutB‐pcr). The four PCR products were combined and fused in the order (*Eco*RI‐left‐pcr, DsRed‐mutA‐pcr, DsRed‐mutB‐pcr, right‐*Sac*II‐pcr) by amplification with the primer pair bn3312/bn3319 to obtain the final PCR fragment *Eco*RI‐left‐DsRed‐right‐*Sac*II, which was sub‐cloned in pJet1.2 resulting in pJ‐*Eco*RI‐left‐DsRed‐right‐*Sac*II. Finally, the sub‐cloned sequences in pJ‐PcUbi‐Cas9 and pJ‐*Eco*RI‐left‐DsRed‐right‐*Sac*II were released with *Spe*I, *Eco*RI and *Eco*RI, *Sac*II, respectively, and inserted in one step in pHEE401E which had been linearized with *Spe*I, *Sac*II, to produce pHEE‐PcUCas9‐35SDsRed. The Golden Gate entry site in pHEE‐PcUCas9‐35SDsRed allows the cloning of several gRNAs. The assembly of gRNA‐scaffolds, TSs and promoters was achieved by PCR following a published protocol (Wang *et al*., 2015). The TSs and the *Bsa*I recognition/restriction sites for Golden Gate cloning were added to the PCR primers as overhangs. The plasmids pCBC‐DT1T2, pCBC‐DT2T3, and pCBC‐DT3T4 containing the gRNA and promoter sequences served as templates (Wang *et al*., 2015; Xing *et al*., 2014).

The gene loci of the camelina accession WT139 were amplified by PCR and re‐sequenced (Table S3), to identify nucleotide exchanges compared to the published genome sequence of the doubled haploid line DH55 (camelina genotype SRS 933; Sequences S1; Kagale *et al*., 2014). TSs for the RNA‐guided Cas9 endonuclease were preselected using the online platforms WU‐CRISPR (Wong *et al*., 2015) and CRIPSPOR (Concordet and Haeussler, 2018), followed by individual *in silico* validation and final selection using criteria as previously outlined (Koeppel *et al*., 2019). The search against the non‐redundant DNA database of *Camelina sativa* at Genbank revealed that the TSs used in the present study are exclusively present in the genes *CsGTR1*, *CsGTR2*, *CsMYB28* and with one mismatch (TS5*) in CsMYB29, diminishing the chances for off‐target effects. For mutagenesis of the homeologs of the *CsGTR1* and *CsGTR2* genes, the respective target‐specific sequences were added as overhangs to the oligonucleotides (Table S4). The primers bn3350 (DT1‐BsF), bn3351 (DT1‐F0) and bn3352 (DT0‐BsR2) were used to amplify the gRNA with target sequence 1—TS1‐pcr; the primers bn3362 (DT2‐BsF2), bn3363 (DT2‐F0) and bn3355 (DT0‐BsR3) were used to amplify the gRNA with target sequence 3—TS3‐pcr; and the primers bn3364 (DT3‐BsF3), bn3365 (DT3‐F0), bn3358 (DT4‐R0) and bn3359 (DT4‐BsR) were used to amplify a PCR product with two gRNAs containing TSs 2 and 4—TS2‐TS4‐pcr. The terms in parentheses correspond to the original primer designations (Wang *et al*., 2015). The order of TSs was modified compared to the published method, because of the high similarity between TSs 3 and 4. The combination of TSs 3 and 4 would have resulted in the failure of the third PCR reaction. The vectors pCBC‐DT1T2, pCBC‐DT2T3, pCBC‐DT3T4 were used as templates for the three PCR reactions TS1‐pcr, TS3‐pcr, and TS2‐TS4‐pcr, respectively. For the Golden Gate cloning, 100 ng of pHEE‐PcUCas9‐35SDsRed and 80 ng of each PCR product (TS1‐pcr, TS3‐pcr, TS2‐TS4‐pcr) were used in a 20 μL reaction mix, supplemented with 0.5 μL ATP (10 mm), BSA (B9000S), T4 DNA ligase buffer and *BsaI*‐HF (R3535S; New England Biolabs, Frankfurt, Germany). After 2 h of incubation at 37 °C, 1 μL of T4 DNA ligase (M0202S) was added followed by incubation at 18 °C for 2 h, 37 °C for 2 h and finally 50 °C for 5 min and 80 °C for 10 min to stop the reactions. The final construct, pHEE‐PcUCas9‐35SDsRed‐GTR1‐GTR2, was transferred into camelina accession WT139 by vacuum infiltration of the flowers with *Agrobacterium tumefaciens* C58 (Lu and Kang, 2008). Transgenic seeds were selected according to their red fluorescence derived from the DsR53ed marker.

For site‐directed mutagenesis of the *CsMYB28* and *CsMYB29* homeologs, three target‐specific sequences were added as overhangs to PCR primers (Table S4). The primers bn3337 (DT1‐BsF), bn3338 (DT1‐F0) and bn3339 (DT0‐BsR2) were used to amplify the gRNA with target sequence 5—TS5‐pcr; and the primers bn3340 (DT2‐BsF2), bn3341 (DT2‐F0), bn3342 (DT3‐R0), bn3343 (DT3‐BsR) were used to amplify a PCR product with two gRNAs containing target motif 6 and 7—TS6‐TS7‐pcr. For the two PCR reactions TS5‐pcr and TS6‐TS7‐pcr, the templates pCBC‐DT1T2 and pCBC‐DT2T3 were used, respectively. The Golden Gate assembly of 100 ng of pHEE‐PcUCas9‐35SDsRed and 80 ng of each PCR product (TS5‐pcr, TS6‐TS7‐pcr) was performed using the same protocol as described above. The resulting construct, pHEE‐PcUCas9‐35SDsRed‐MYB28‐MYB29, was employed for the transformation of camelina.

### Glucosinolate measurements

Glucosinolates were extracted following a published protocol with modifications (Doheny‐Adams *et al*., 2017). Ten camelina seeds which had been dried under vacuum for 48 h were ground in 1 mL 80% methanol and 20 μL of internal standard (4 mm sinigrin in methanol) with ceramic beads in a Precellys homogenizer (Bertin Technologies, Montigny‐le‐Bretonneux, France). The sample was centrifuged and the glucosinolates in the supernatant purified via ion exchange chromatography. The sample was loaded on a column containing 0.1 g Sephadex (DEAE A25120) soaked before overnight in 2 mL 0.5 m acetic acid buffer (pH 5 with NaOH). The resin with the bound glucosinolates was washed five times with one column volume of water and three times with 0.02 m acetic acid buffer (pH 5 with NaOH). Subsequently, 500 μL of a solution containing 1 U of sulfatase (type H‐1; Merck/Sigma‐Aldrich, Taufkirchen, Germany) in 0.02 m acetic acid buffer (pH 5, NaOH) was loaded on the column, the column sealed after the flow of three drops of buffer and incubated overnight. The desulfoglucosinolates were eluted with 2 mL of water, dried in a vacuum concentrator, dissolved in 200 μL of water and measured by HPLC. This protocol was also applied for glucosinolate extraction from embryo and seed coat samples.

Prior to extraction of glucosinolates from silicle walls (carpels, valves) they were freeze dried and ground in a mortar. About 250–400 mg of ground material was subjected to extraction with 80% methanol at 60 °C for 4 h in the presence of 25 μL 4 mm sinigrin as internal standard. Columns with 0.6 g Sephadex were prepared and loaded with the glucosinolate extract and proceeded as described for seeds and incubated over night with 3 mL of a solution with 3 U of the sulfatase. The desulfoglucosinolates were eluted, dried and dissolved in 100 μL of HPLC grade water and measured with HPLC.

Glucosinolates were extracted from roots with 40 mL of 80% methanol at 80 °C for 12 h in the presence of the internal standard (40 μL of 4 mm sinigrin). Sephadex resin (1.0 g) was soaked overnight in 10 mL acetic acid buffer (0.5 m), centrifuged and the buffer was removed. Glucosinolates were bound to the Sephadex resin and purified in a batch procedure in 50 mL Falcon tubes. The resin was washed with 30 mL of water or acetic acid buffer (0.02 m) as described above, and water or buffer were removed after each step by centrifugation. Finally, the Sephadex resin with the bound glucosinolates was incubated overnight with 5 mL of a solution containing 3 U of sulfatase (see above). The desulfoglucosinolates were eluted in 30 mL of water followed by centrifugation to remove the Sephadex resin. The sample was concentrated in a rotary evaporator and the desulfoglucosinolates dissolved with 200 μL of water.

Glucosinolates were measured by HPLC following a published procedure (Doheny‐Adams *et al*., 2017). The glucosinolates were separated on a Eurospher II 100‐3 C18 A column with pre‐column, using a linear gradient (buffer A, H_2_O; buffer B, H_2_O/acetonitrile, 70:30, v/v) and detected by UV absorption at 229 nm. The flow rate was 0.4 mL/min with 5 μL injection volume (Doheny‐Adams *et al*., 2017). The response factors for sinigrin, glucoarabin/GL9, glucocamelinin/GL10 and gluconesliapaniculatin/GL11 were taken from published values (Grosser and van Dam, 2017; Lange *et al*., 1995).

### Seed fatty acid and protein measurements

Fatty acid analyses of seeds (three seeds per sample) were performed after converting the lipids into fatty acid methyl esters which were quantified by GC (Browse *et al*., 1986). Total proteins were extracted from three seeds per sample and quantified with the Lowry DC protein assay (Bio‐Rad; Hölzl and Dörmann, 2021).

### Expression analysis by qPCR

Total RNA was extracted from camelina WT roots, whole green seeds (embryo stage) and fully developed green embryos using the QIAzol reagent (Qiagen, Hilden, Germany). After digestion of genomic DNA with RNase‐free DNase I (Thermo Fisher Scientific), cDNA was synthesized by reverse transcription using the First Strand cDNA Synthesis Kit (Thermo Fisher Scientific). α‐Tubulin from camelina was used as housekeeping gene (Frerigmann and Gigolashvili, 2014; Stracke *et al*., 2001). Primers and the respective locus identifiers are provided in Table S5.

### Accession numbers

The following sequences were included in this study: CsGTR1‐A, B, C, LOC104711273, LOC104791006, LOC104780607; CsGTR2‐A, B, C, LOC104762400, LOC104726867, LOC104740346; CsMYB28A, B, C, LOC104726729, LOC104762254, LOC104738807; CsGTR3‐A, B, C, LOC104740588, LOC104776006, LOC104756232; CsMYB29‐A, B, C, LOC104734792, LOC104768979, LOC104708326.

## Conflict of interest

The authors declare no conflict of interest.

## Author contributions

G.H. and P.D. designed the experiments and wrote the manuscript. G.H. managed plant care in the greenhouse and growth chambers. G.H., B.R. and J.K. selected the target sequences and designed and constructed the vectors. G.H. transformed camelina plants and screened and analysed plants. All authors provided technical suggestions.

## Supporting information


**Figure S1** The binary vectors used for plant transformation.
**Figure S2** Mutant analysis by restriction digest of PCR products amplified from the *CsGTR1* and *CsGTR2* homeologs.
**Figure S3** Mutation pattern of *csgtr1csgtr2* mutants.
**Figure S4** Mutation pattern of *csmyb28csmyb29* mutants.
**Figure S5** Fatty acid profiles of (a) *csgtr1csgtr2* (G1, G2, G3), and (b) *csmyb28csmyb29* (M1–M5) mutant lines compared to wild type (WT).
**Table S1** Oligonucleotides used for the amplification of the region containing the target sequences of the three homeologs (A, B, C) each of *CsGTR1*, *CsGTR2*, *CsMYB28* and *CsMYB29*.
**Table S2** Oligonucleotides used for the amplification of DNA fragments for modification of pHEE401E.
**Table S3** Oligonucleotides used for the PCR amplification of the homeologs (A, B, C) each of *CsGTR1*, *CsGTR2* and *CsMYB28*, and for sequencing of the WT genes in accession WT139.
**Table S4** Oligonucleotides used for the amplification of the gRNA sequences containing the target sequences.
**Table S5** Oligonucleotides used for qPCR analysis of the homeologs (A, B, C) each of *CsGTR1*, *CsGTR2*, *CsMYB28* and *CsMAM1*.
**Sequences S1** Sequence analyses of the three homeologous genes of each *CsGTR1*, *CsGTR2*, C*sMYB28* and *CsMYB29* in *Camelina sativa* accession WT139.
