# Supplementary material for: Ablation of glucosinolate accumulation in the oil crop Camelina sativa by targeted mutagenesis of genes encoding the transporters GTR1 and GTR2 and regulators of biosynthesis MYB28 and MYB29
Source: Plant Biotechnol J. 2022 Oct 14;21(1):189–201. doi: 10.1111/pbi.13936 (PMC9829395; doi:10.1111/pbi.13936)
Supplement: Supplementary file 1 — Figure S1 The binary vectors used for plant transformation. Figure S2 Mutant analysis by restriction digest of PCR products amplified from the CsGTR1 and CsGTR2 homeologs. Figure S3 Mutation pattern of csgtr1csgtr2 mutants. Figure S4 Mutation pattern of csmyb28csmyb29 mutants. Figure S5 Fatty acid profiles of (a) csgtr1csgtr2 (G1, G2, G3), and (b) csmyb28csmyb29 (M1–M5) mutant lines compared to wild type (WT). Table S1 Oligonucleotides used for the amplification of the region containing the target sequences of the three homeologs (A, B, C) each of CsGTR1, CsGTR2, CsMYB28 and CsMYB29. Table S2 Oligonucleotides used for the amplification of DNA fragments for modification of pHEE401E. Table S3 Oligonucleotides used for the PCR amplification of the homeologs (A, B, C) each of CsGTR1, CsGTR2 and CsMYB28, and for sequencing of the WT genes in accession WT139. Table S4 Oligonucleotides used for the amplification of the gRNA sequences containing the target sequences. Table S5 Oligonucleotides used for qPCR analysis of the homeologs (A, B, C) each of CsGTR1, CsGTR2, CsMYB28 and CsMAM1. Sequences S1 Sequence analyses of the three homeologous genes of each CsGTR1, CsGTR2, CsMYB28 and CsMYB29 in Camelina sativa accession WT139. [file PBI-21-189-s001.docx]

**SUPPORTING INFORMATION**

**Ablation of glucosinolate accumulation in the oil crop *Camelina sativa* by targeted mutagenesis of genes encoding the transporters GTR1 and GTR2 and regulators of biosynthesis MYB28 and MYB29**

Georg Hölzl^1^, Barno Rezaeva^2^, Jochen Kumlehn^2^ and Peter Dörmann^1,^*

^1^Institute of Molecular Physiology and Biotechnology of Plants, University of Bonn, Karlrobert-Kreiten-Strasse 13, D-53115 Bonn, Germany

^2^Leibniz Institute of Plant Genetics and Crop Plant Research (IPK) Gatersleben, Plant Reproductive Biology, Corrensstrasse 3, D-06466 Seeland, OT Gatersleben, Germany

*Corresponding author.

E-mail address: doermann@uni-bonn.de

(Tel +49-73-2830; fax +49-73-1696)

**
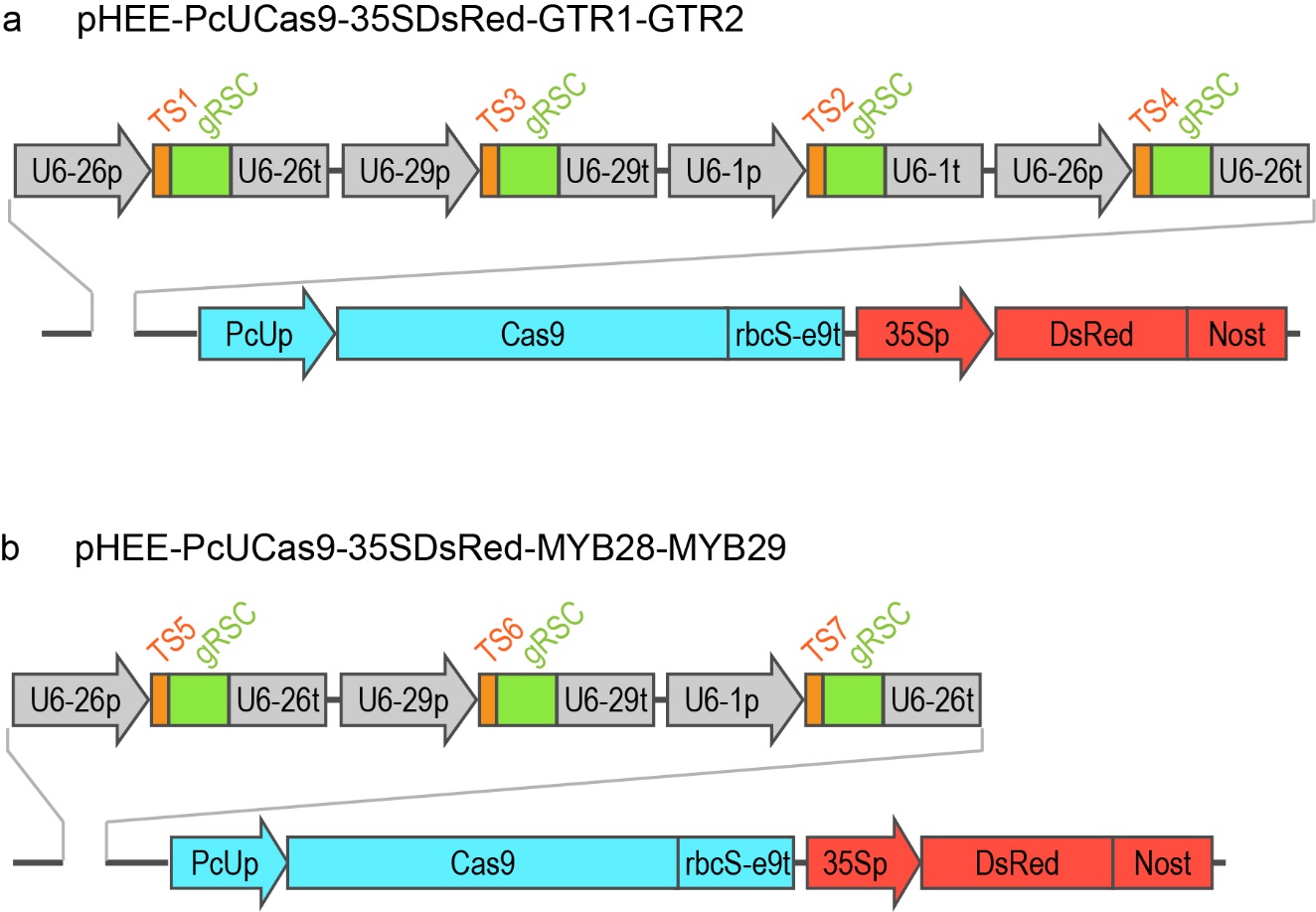
**

**Figure S1 The binary vectors used for plant transformation.** The vectors contain the cas9 endonuclease expression cassette (blue) with ubiquitin promoter (PcUp) from parsley and the rbcS E9 terminator (rbcS-e9t) from pea. The selection marker DsRed (red bar) is expressed under control of the 35S promoter (35Sp) and the NOS terminator (Nost). The expression of the single guide RNAs (gRNA) consisting of the 20 bp target sequences (TS1-4 or TS5-7, orange) plus the gRNA scaffolds (gRSC, green) was under control of the polymerase III-processed U6-26, U6-29, U6-1 promoters and the respective terminators (grey) from Arabidopsis. (a) pHEE-PcUCas9-35SDsRed-GTR1-GTR2; (b) pHEE-PcUCas9-35SDsRed-MYB28-MYB29.

**
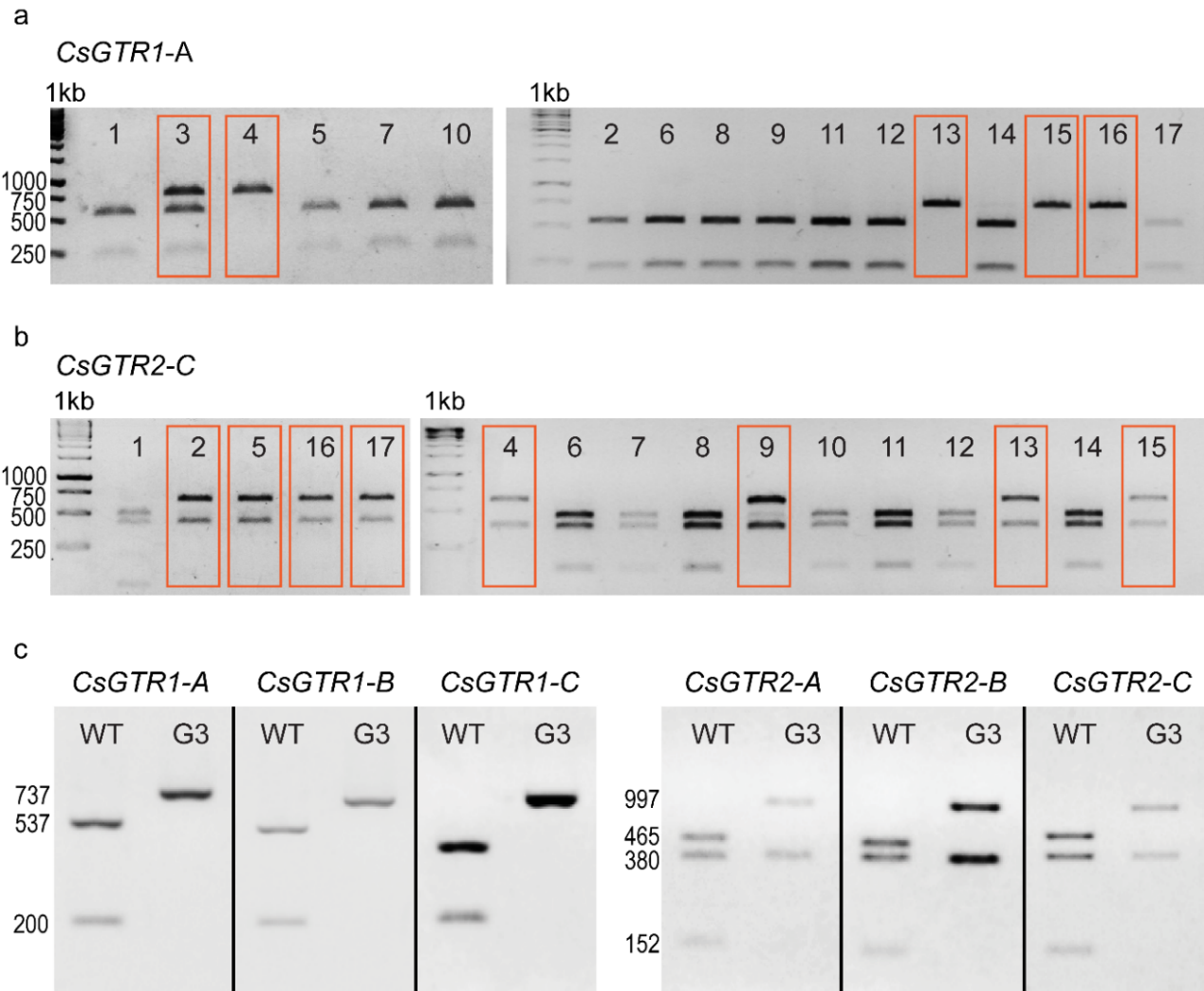
**

**Figure S2 Mutant analysis by restriction digest of PCR products amplified from the *CsGTR1* and *CsGTR2* homeologs.** Target sequences of the different homeologs were amplified with specific primer pairs and the PCR products were digested with *Tsp45*I. The *Tsp45*I restriction site is present in TS1 which is found in all homeologs of *CsGTR1* and *CsGTR2*. Disruption of the restriction site by mutagenesis might prevent digestion. A further *Tsp45*I restriction site is found in the three homeologs of *CsGTR2* downstream of TS1, leading to an additional fragment after digestion. (a) and (b) Analysis of *cas9*-transgenic T1 lines (lines 1-17) for mutations in *CsGTR1*-A and *CsGTR2*-C. Mutagenized sequences are highlighted with orange boxes. Note that samples 3 and 9 carry a WT and mutant allele in *CsGTR1-A* or *CsGTR2-C*. (c) Analysis of the T-DNA-free T2 line G3 (descendant of line 16) indicates the complete mutagenesis of all *CsGTR1* and *CsGTR2* homeologs. Digestion of the PCR products from *CsGTR1-A* (737 bp) results in two bands with 537 and 200 bp, and from *CsGTR2-C* (997 bp) leads to 3 bands with 465, 380 and 152 bp. The fragment with 380 bp is derived from a second *Tsp45*I restriction site present in all *CsGTR2* homeologs. The digestion of PCR products of *CsGTR1-B* and *CsGTR1-C* results in similar sizes compared to *CsGTR1-A*, and digestion of *CsGTR2-A* and *CsGTR2-B* homeologs results in similar sizes as shown for *CsGTR2-C*. The distances between the bands of the corresponding homeologs vary because the DNA fragments were separated on different gels.


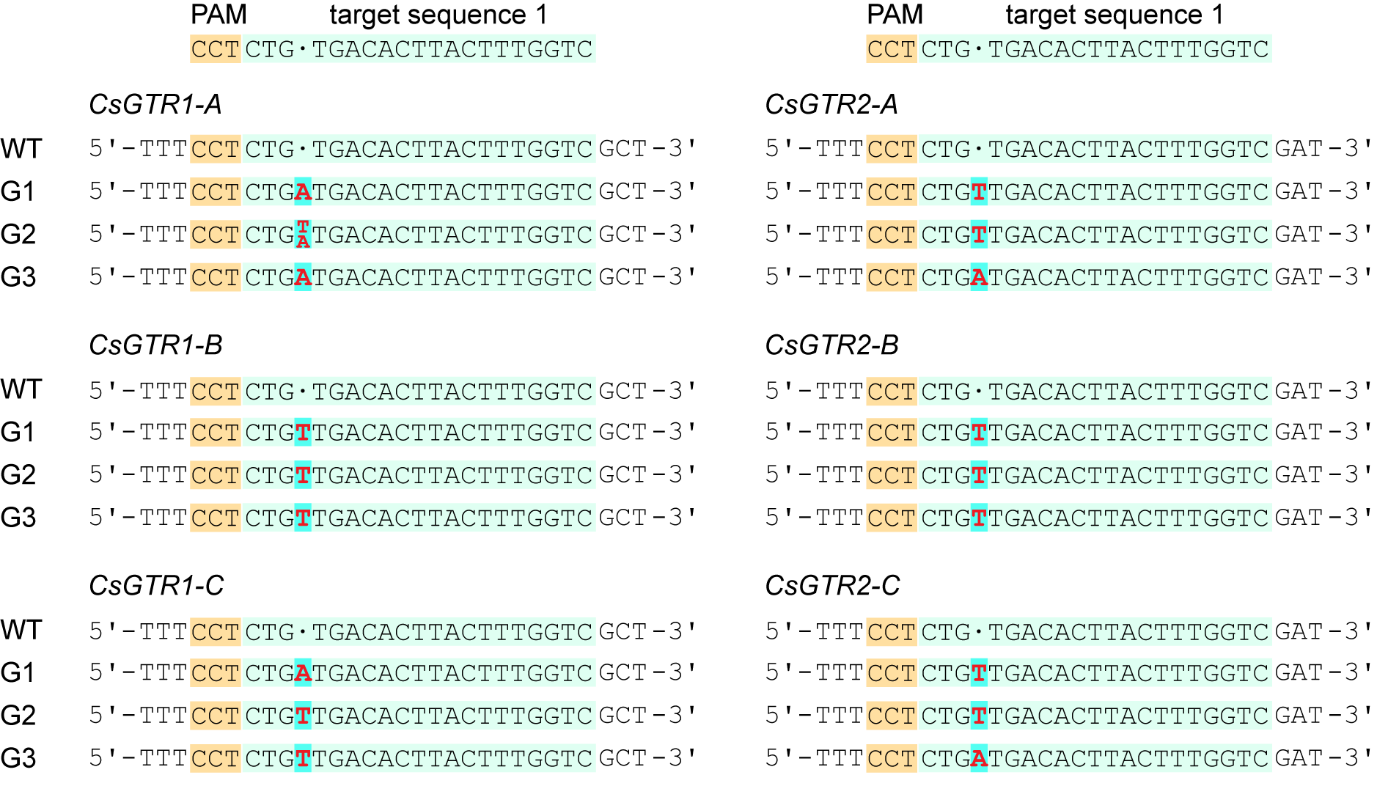


**Figure S3 Mutation pattern of *csgtr1csgtr2* mutants.** The plants with homozygous mutations were obtained in the T2 generation. A and T insertions are found in the target sequence TS1 of the three homeologs of *CsGTR1* and *CsGTR2* (A, B, C) in the lines G1, G2 and G3. All mutations are homozygous with one exception, i.e. *CsGTR1-A* in line G2 which carries a biallelic mutation (T/A).

| 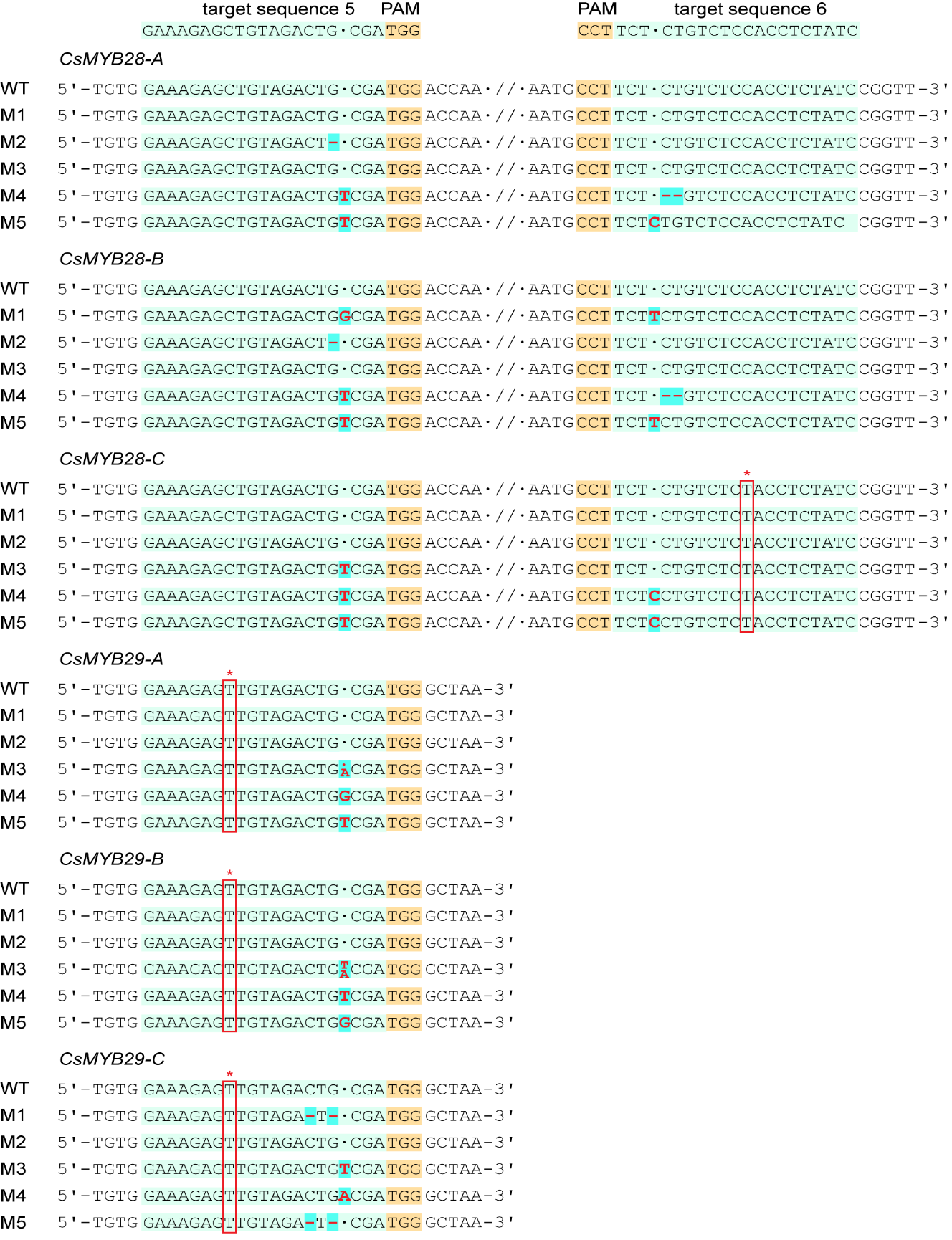 |
| --- |

**Figure S4 Mutation pattern of *csmyb28csmyb29* mutants.** The plants with homozygous insertions or deletions in TS5 and TS6 of the three homeologs of *CsMYB28* (A, B, C) were obtained in the T3 generation in the lines M1-M5. Mutations were also detected in *CsMYB28-C* in the TS6* sequence (red box) with 1 bp difference to TS6. Besides, mutations were observed in *CsMYB29* (A, B, C) in the lines M1, M3, M4, M5 in the TS5* sequence (red box) with 1 bp difference to TS5. All mutations were homozygous except for *CsMYB29-A* and *CsMYB29-B* of line M3 being heterozygous (∙/A) or biallelic (T/A), respectively.

*
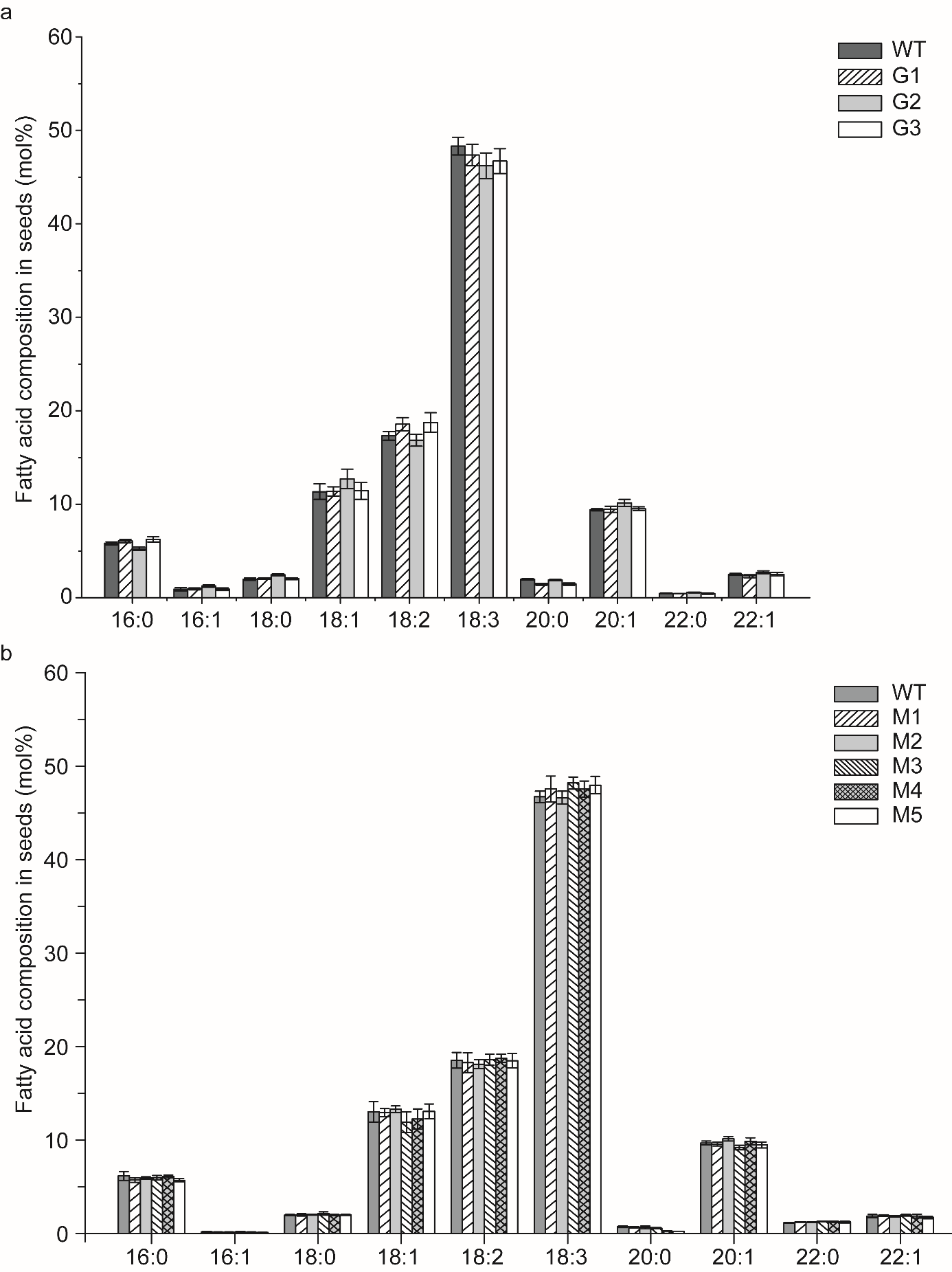
*

**Figure S5 Fatty acid profiles of (a) *csgtr1csgtr2* (G1, G2, G3), and (b) *csmyb28myb29* (M1-M5) mutant lines compared to wild type (WT).** Fatty acids were measured as methyl esters by GC. The seed fatty acid profiles correspond to the seed lipid measurements in Figure 5a, 5b, with three seeds pooled per measurement, n = 5.

**Table S1** **Oligonucleotides used for the amplification of the region containing the target sequences of the three homeologs (A, B, C) each of *CsGTR1*, *CsGTR2,* *CsMYB28* and *CsMYB29*.** These PCR products comprise only the target sequences and were used for mutant screening by restriction digest and sequencing.

| Name | Oligonucleotide sequence 5’→3’ | Size (bp) | Locus |
| --- | --- | --- | --- |
|  | *CsGTR1* |  |  |
| Bn3439 | gagaaggtatctttatcatattt | 737 | LOC104711273 (A) |
| Bn3440 | TAACTTTTACATAGAGTCTATCC |  |  |
| Bn3441 | tatgagaaggtagtattctttg | 623 | LOC104791006 (B) |
| Bn3432 | GAGTGAGACGATCTGCGCA |  |  |
| Bn3442 | tgcaataaatactttctaacttc | 661 | LOC104780607 (C) |
| Bn3443 | GACTGGATGTACACAATTAGC |  |  |
|  | *CsGTR2* |  |  |
| Bn3049 | TTTCTTCTTTTTAATTTGTTGTTG | 1051 | LOC104762400 (A) |
| Bn3445 | GATGCAGTTTTGGCACTGCT |  |  |
| Bn3053 | CTATTTTCTTCTTTTTTGTTGTTG | 1035 | LOC104726867 (B) |
| Bn3446 | TTTGGCACTGCAGCAGTCAT |  |  |
| Bn3057 | GACTATTTTCTTCTTTTGTTGTA | 1000 | LOC104740346 (C) |
| Bn3447 | AAACGATCCCTACAAAAACCAT |  |  |
|  | *CsMYB28* |  |  |
| Bn3402 | tacatccacgaccacggc | 952 | LOC104726729 (A) |
| Bn3242 | TAGAACTATCCTCTTCACTGCA |  |  |
| Bn3403 | ctacatccacgaccacggt | 948 | LOC104762254 (B) |
| Bn3244 | GAACTATCCTCTTCACTGCGG |  |  |
| Bn3404 | ggtgaaggaggctggcgt | 936 | LOC104738807 (C) |
| Bn3246 | TCGTCTTCACTGCGAATTTGC |  |  |
|  | *CsMYB29* |  |  |
| Bn4758 | gtttgttgttttagcgaagg | 640 | LOC104734792 (A) |
| Bn4759 | GATCGATTCCTTGATCGATC |  |  |
| Bn4760 | ataccaccagtccaccacc | 649 | LOC104768979 (B) |
| Bn4761 | GATCGATTCCCTGATCGGTA |  |  |
| Bn4762 | gtacttaattatactcctatg | 664 | LOC104708326 (C) |
| Bn4763 | ATCGATTCCCTGATCGACC |  |  |

**Table S2 Oligonucleotides used for the amplification of DNA fragments for modification of pHEE401E**. The PCR fragment from plasmid pHEE401E from Addgene (plasmid # 71287; http://n2t.net/addgene:71287; RRID:Addgene_71287) was used to produce the vector pHEE-PcUCas9-35SDsRed. Restriction sites are underlined. Overhangs added to the primers for fusion of overlapping PCR products and the mutagenized *Bsa*I site (present in the original DsRed expression cassette) are underlined. The nucleotide of the mutagenized position is depicted in red.

| Name | Oligonucleotide sequence 5’→3’ | Restriction site |
| --- | --- | --- |
| Bn3308 | TTTACTAGTGAATCCAAAAATTACGGATATG | *Spe*I |
| Bn3309 | TTTGGTACCCTGCACATACATAACATATCAAG | *Kpn*I |
|  |  |  |
| Bn3310 | TTTGGTACCATGGATTACAAGGACCACGAC | *Kpn*I |
| Bn3311 | TTTGAATTCGTTGTCAATCAATTGGCAAGTC | *Eco*RI |
|  |  |  |
| Bn3312 | TTTGAATTCGTAATCATGTCATAGCTGTTTC | *Eco*RI |
| Bn3313 | CTACATCTTGGATAATTACCTTATCACATCAATCCACTTGCTTT |  |
|  |  |  |
| Bn3314 | AGCAAGTGGATTGATGTGATAAGGTAATTATCCAAGATGTAGCA |  |
| Bn3315 | CTTCAGGGCCTTGTGGGTTTCGCCCTTCAGCAC |  |
|  |  |  |
| Bn3316 | GGCGTGCTGAAGGGCGAAACCCACAAGGCCCTGAAGC |  |
| Bn3317 | GGGGGATCTGGATTTTAGTACTAACATAGATGACACCGCGCG |  |
|  |  |  |
| Bn3318 | CGCGCGGTGTCATCTATGTTAGTACTAAAATCCAGATCCCCC |  |
| Bn3319 | TTTCCGCGGTGATCACAGGCAGCAACGC | *Sac*I |

**Table S3** **Oligonucleotides used for the PCR amplification of the homeologs (A, B, C) each of *CsGTR1*, *CsGTR2* and *CsMYB28*, and for sequencing of the WT genes in cultivar WT139.** The homeologs of *CsGTR1* and *CsGTR2* were amplified as two overlapping PCR products (PCR1, PCR2).

| Name | Oligonucleotide sequence 5’→3’ | Size (bp) | Locus |
| --- | --- | --- | --- |
|  | *CsGTR1* |  |  |
| Bn3037 | CTAGATCCACACACACCAC | 1567 | LOC104711273 (A), PCR1 |
| Bn3038 | AACTTTTACATAGAGTCTATCC |  |  |
| Bn3039 | AGATCATCTCACTCACACTC | 1402 | LOC104711273 (A), PCR2 |
| Bn3040 | TTTAATCTATATAAAGTACAACAG |  |  |
| Bn3306 | TAAGGAAGACCGAAGCATAG | 1851 | LOC104791006 (B), PCR1 |
| Bn3307 | CTAGAAACCTGACAACACATG |  |  |
| Bn3043 | TTCACGTTTGCGCAGATCG | 1356 | LOC104791006 (B), PCR2 |
| Bn3044 | AAGAAACTGAGGTCGTTGAC |  |  |
| Bn3045 | CGCTAGATCCACACCACTA | 1684 | LOC104780607 (C), PCR1 |
| Bn3046 | GCTTAACAGGCTTCAATCCT |  |  |
| Bn3047 | CACTCACGCTAATTGTGTAC | 1404 | LOC104780607 (C), PCR2 |
| Bn3048 | TTTCTTCATTTTAATCTATATAAAG |  |  |
|  | *CsGTR2* |  |  |
| Bn3049 | TTTCTTCTTTTTAATTTGTTGTTG | 1944 | LOC104762400 (A), PCR1 |
| Bn3050 | CGATGAAGATTGTCATTCCG |  |  |
| Bn3051 | TGCTTGATTTTCTTTGCGGGA | 1225 | LOC104762400 (A), PCR2 |
| Bn3052 | TTCCAGAAGATGAGAGAAACA |  |  |
| Bn3070 | TTGTTGTTGTAATAATTTACTTGT | 1907 | LOC104726867 (B), PCR1 |
| Bn3071 | ATTCCTGTCATCAAGAAAACC |  |  |
| Bn3055 | CTTTTGGTGCTGATCAGTTC | 1413 | LOC104726867 (B), PCR2 |
| Bn3056 | TTCCAGAAGATGAGAGAAACA |  |  |
| Bn3057 | GACTATTTTCTTCTTTTGTTGTA | 1833 | LOC104740346 (C), PCR1 |
| Bn3058 | GAAGACGGGGTAAGTCATC |  |  |
| Bn3059 | ACTCTAGTCGTGTACATTCAA | 1308 | LOC104740346 (C), PCR2 |
| Bn3060 | CTTCCAGAAGATGAGAAACC |  |  |
|  | *CsMYB28* |  |  |
| Bn3031 | CTTATCAAGTTAAACAGTATCC | 1455 | LOC104726729 (A) |
| Bn3032 | TTGTCTTGCGGATATATATCC |  |  |
| Bn3033 | CTTATCTAGTTGAACAATATCC | 1367 | LOC104762254 (B) |
| Bn3034 | ATACTAGATACATACACAACAC |  |  |
| Bn3035 | TCTTTCTTATCAAGTTAAATTCC | 1349 | LOC104738807 (C) |
| Bn3036 | GCGAGTTATAGTAAATGACAAG |  |  |

**Table S4 Oligonucleotides used for the amplification of the gRNA sequences containing the target sequences.** TS1 is highlighted in light green, TS3 in yellow, TS2 in pink, TS4 in light blue, TS5 in dark red, TS6 in dark blue and TS7 in dark green. TS1 to TS4 are found in *CsGTR1* and/or *CsGTR2*, TS5 to TS7 are found in *CsMYB28,* and TS5* with one bp difference in *CsMYB29*. *Bsa*I recognition sites are shown in red and 5' protruding ends produced by *Bsa*I digestion are underlined. The published primer designations^1^ are indicated in the right column.

| Name | Oligonucleotide sequence 5’→3’ | Published Name^1^ |
| --- | --- | --- |
| *CsGTR1*, *CsGTR2* | | |
| Bn3350 | ATATATGGTCTCGATTGACCAAAGTAAGTGTCACAGGTT | DT1-BsF |
| Bn3351 | TGACCAAAGTAAGTGTCACAGGTTTTAGAGCTAGAAATAGC | DT1-F0 |
| Bn3352 | ATATTATTGGTCTCAATCTCTTAGTCGACTCTACCAAT | DT0-BsR2 |
|  |  |  |
| Bn3362 | ATATTATTGGTCTCAAGATTGGAGGAAGGGTTTTGGTCCTGTT | DT2-BsF2 |
| Bn3363 | TGGAGGAAGGGTTTTGGTCCTGTTTTAGAGCTAGAAATAGC | DT2-F0 |
| Bn3355 | ATATTATTGGTCTCATCACTACTTCGTCTCTAACCAT | DT0-BsR3 |
|  |  |  |
| Bn3364 | TATTATTGGTCTCAGTGATTGGAAACAAAAGCACATGCGAGTT | DT3-BsF3 |
| Bn3365 | TGGAAACAAAAGCACATGCGAGTTTTAGAGCTAGAAATAGC | DT3-F0 |
| Bn3358 | AACAGGACCAAAACTCTTCCTCCAATCACTACTTCGACTCTAGCTGTAT | DT4-R0 |
| Bn3359 | ATTATTGGTCTCTAAACAGGACCAAAACTCTTCCTCC | DT4-BsR |
|  |  |  |
| *CsMYB28, CsMYB29* | | |
| Bn3337 | ATATATGGTCTCGATTGAAAGAGCTGTAGACTGCGAGTT | DT1-BsF |
| Bn3338 | TGAAAGAGCTGTAGACTGCGAGTTTTAGAGCTAGAAATAGC | DT1-F0 |
| Bn3339 | ATATTATTGGTCTCAATCTCTTAGTCGACTCTACCAAT | DT0-BsR2 |
|  |  |  |
| Bn3340 | ATATTATTGGTCTCAAGATTGATAGAGGTGGAGACAGAGAGTT | DT2-BsF2 |
| Bn3341 | TGATAGAGGTGGAGACAGAGAGTTTTAGAGCTAGAAATAGC | DT2-F0 |
| Bn3342 | AACCCTTGCATTGGTGTTCCATCAATCACTACTTCGTCTCTAACCAT | DT3-R0 |
| Bn3343 | ATTATTGGTCTCGAAACCCTTGCATTGGTGTTCCATC | DT3-BsR |

^1^ Wang, Z.-P., Xing, H.-L., Dong, L., Zhang, H.-Y., Han, C.-Y., Wang, X.-C. and Chen, Q.-J. (2015) Egg cell-specific promoter-controlled CRISPR/Cas9 efficiently generates homozygous mutants for multiple target genes in Arabidopsis in a single generation. Genome Biol. 16, 144.

**Table S5 Oligonucleotides used for qPCR analysis of the homeologs (A, B, C) each of *CsGTR1*, *CsGTR2*, *CsMYB28* and *CsMAM1*.** The oligonucleotides for amplifying the housekeeping (*α-tubulin*) and the *CsMAM1* genes bind in regions conserved in all three homeologs. Two different primer pairs were used to amplify the housekeeping gene. The PCR product of bn3996/bn3997 served as reference for *CsGTR1*, *CsGTR2* and *CsMYB28*, and the PCR product of bn4548/bn4549 served as reference for *CsMAM1*.

| Name | Oligonucleotide sequence 5’→3’ | Locus |
| --- | --- | --- |
|  | *CsGTR1* |  |
| Bn3995 | CGGCTTCAGATCCATGGAAA | LOC104711273 (A) |
| Bn3304 | TTATTGCAAGATAGTAAATCGAG |  |
| Bn3261 | TTCCGCAGCTAACACTTGCT | LOC104791006 (B) |
| Bn3262 | AGATAGAACCGGCGAAGCTT |  |
| Bn3251 | CAATAAGTATGCAAATGACTTAC | LOC104780607 (C) |
| Bn3525 | TACATAGGTTGCTCCGGGG |  |
|  | *CsGTR2* |  |
| Bn3253 | CCATAACTCAACAAATGACTTAT | LOC104762400 (A) |
| Bn3254 | AACGTAGGTGGCAGCAGGA |  |
| Bn3526 | GCTGCATGTGGAACAGCAGT | LOC104726867 (B) |
| Bn3527 | TCTGATCCCACCTGCTCCC |  |
| bn3995 | GAAGAAGTGAAGTGCATTGTG | LOC104740346 (C) |
| bn3996 | GGAAGACGGGGTAAGTCATC |  |
|  | *CsMYB28* |  |
| Bn3241 | AAGGATATATTGTCGGCTTCCA | LOC104726729 (A) |
| Bn3242 | TAGAACTATCCTCTTCACTGCA |  |
| Bn3523 | GCAAGAAAAATTTCAAGAAATCG | LOC104762254 (B) |
| Bn3524 | GCACTTAAGCTACCTTCCAC |  |
| Bn3245 | GCTAAGGCCACTTCCATCAAA | LOC104738807 (C) |
| Bn3246 | TCGTCTTCACTGCGAATTTGC |  |
|  | α-Tubulin |  |
| Bn3996 | CACCCTGAGCAACTCATCAG | LOC104703779  LOC104758270  LOC104777925 |
| Bn3997 | TCCAAGCACAGATCAACAATC |  |
| Bn4548 | Gtggtcattacaccattggg |  |
| Bn4549 | CAACAGCGTTGAAGACAAGGA |  |
|  | *CsMAM1* |  |
| Bn4546 | AAGGATATATTGTCGGCTTCCA | LOC104770650  LOC104706601  LOC104736315 |
| Bn4547 | TAGAACTATCCTCTTCACTGCA |  |

**Supplementary Sequences**

**Sequence analyses of the three homeologous genes of each *CsGTR1*, *CsGTR2, CsMYB28* and *CsMYB29* in *Camelina sativa* accession WT139**

Oligonucleotides were designed based on the published sequences (locus identifiers). Genomic DNA from camelina wild type (WT139) was used as template for PCR amplification and sequencing. The sequences obtained for cultivar WT139 (see below) differ slightly from the published sequences. Base exchanges between the published cultivar and WT139 are highlighted in light blue, insertions are underlined, exons are labelled in gray, and start and stop codons are shown in bold letters. Between the start and stop codons, five base exchanges were found in *CsGTR1-A*, one in *CsGTR1-*B, two in *CsGTR1-*C, one in *CsGTR2-A*, eight in *CsGTR2-*B, one in *CsGTR2-*C, two in *CsMYB28-A*, two in *CsMYB28-B*, and 10 in *CsMYB28-C,* which in addition contained 10 insertions. Sequences from GenBank are shown for the three loci of *CsMYB29* (*CsMYB29-A, CsMYB29-B, CsMYB29-C*).

Based on the sequencing data, 4 target sequences were identified for *CsGTR1* and *CsGTR2* (TS1 to TS4) and 3 target sequences (TS5, TS6, TS7) for *CsMYB28* while TS5* matches to *CsMYB29* with one bp mismatch. Target sequences are highlighted in different colors, and the PAM sequences are underlined.

| Gene | Locus identifiers |
| --- | --- |
| *CsGTR1-A* | LOC104711273 |
| *CsGTR1-B* | LOC104791006 |
| *CsGTR1-C* | LOC104780607 |
| *CsGTR2-A* | LOC104762400 |
| *CsGTR2-B* | LOC104726867 |
| *CsGTR2-C* | LOC104740346 |
| *CsMYB28-A* | LOC104726729 |
| *CsMYB28-B* | LOC104762254 |
| *CsMYB28-C* | LOC104738807 |
| *CsMYB29-A* | LOC104734792 |
| *CsMYB29-B* | LOC104768979 |
| *CsMYB29-C* | LOC104708326 |

TS1: GACCAAAGTAAGTGTCACAGAGG

TS2: GGAAACAAAAGCACATGCGAAGG

TS3: GGAGGAAGGGTTTTGGTCCTTGG

TS4: GGAGGAAGAGTTTTGGTCCTTGG

TS5: GAAAGAGCTGTAGACTGCGATGG

TS5*: GAAAGAGTTGTAGACTGCGATGG

TS6: GATAGAGGT**G**GAGACAGAGAAGG

TS6*: GATAGAGGT**A**GAGACAGAGAAGG

TS7: GATGGAACACCAATGCAAGGCGG

**CsGTR1-A**

ACACACCACTATATAAGGAAGACCGAATCATAGAG**ATG**AAGAGCAGAGTCATCCTTAACC

ATAGAGACAAAAGAGATAAGAACAATAACAACAACAACAGCGACCTCTTCTGTAATTACA

CACAGATTGAGACTATGGAGAGAAAGCCCCTTGAGGTTGAGACGACGACAACGAACCACC

ACTCCGCCGTCGATGATGTGACATCTACGACGGTCGATTCATTCGAGGAAGATCAGAAAA

AACTTGTTTATAGAGGATGGAAAGTCATGCCCTTCATCATTGGTAAACTCACCCTTTTGT

TTTTCTCTTTCGGACATATATATTCTTTTGTTGTGTTTTGTCGGAATTTTAGAAAAAAAA

AATGAAATATTCTGTAAAACATTTTGTCCGTGATATTATTAAATTTTCGTTTTCTCTTTT

TTTTACTTGTCTTAACTCTTAAGACTTGACTTGTATCAAAAAAATCTCGACTTTGGACGA

AAAAAATGGTTTAGCAAAATATAGATTTGGATGAAACCCAGATGATATGTAAGATCTATC

TATGAAAAAAAAGATAGTTTTTTTTCAAAAAAAAATTAATTTTCCGTAGAAAAAAAGAGA

CTTTGAATCAAGCAGTTAAGTTTCTGAAAAAAAAAGTAGATAAGAATTTTGACATTTTTC

TGGATAAGACAAAAAATGATTAACTTCTGGGAGTTACAGAGCACTTAGAACCAAAAAAAA

AAGTCAGAACATCATCGAAAGTCCAATCTTTTGACTTGTAGGTTGTGTGTTTCGGTTTCG

AAGTTGTAACATTAAATGCAATAAATACTTTGTAACTTTATGAGAAGGTATCTTTATCAT

ATTTATGTGGTGCTGTTGACAGGTAATGAGACATTTGAGAAGCTTGGGATCATAGGAACA

TTATCGAACCTTCTTGTCTACCTAACTTCTGTATTCAACCTTAAGAGCTATACAGCTGCA

ACTATCATCAATGCCTTTAGTGGCACTATCAATTTCGGCACTTTCATTGCTGCTTTCCTC

TGTGACACTTACTTTGGTCGCTACAAGACTCTCTGTGTCGCTGTCATCGCTTGTTTCATG

GTACTTTTATATATAATCTTTGATGAGTTAACTTGGTTTTGTATTTCCCAATATTTGTGT

AAAACATTTTCATTTTTGTAGGGATCGTTTGTGATACTACTGACTGCTGCAATTCCTTCA

TTGCACCCCGTTGCTTGCGGAAACAAAAGCACATGCGAAGGTCCAAGCGTGGGACAGATA

ATGTTTCTGCTCATGGGTTTAGGGTTTCTTGTGATAGGCGCTGGTGGTATCAGACCGTGT

AATTTAGCGTTTGGTGCTGATCAGTTCAACCCCAAATCCGAATCAGGAAAGAAAGGGATC

AACAGTTTCTTCAACTGGTATTTCTTCACGTTCACGTTCGCTCAGATCATCTCACTCACA

CTCATTGTGTATATCCAGTCAAATGTGAGTTGGACCATCGGTTTGACTATCCCTGTGGTT

CTTATGTTCTTGGCCTGCGTCATCTTCTTTGCTGGGGATAGACTCTATGTAAAAGTTAAA

GCCTCGGGTAGTCCTTTGGCTGGTATAGCTCATGTTATAGCAGCCGCTATCAAGAAACGT

GGTTTGAAGCCTGTTAAGCAGCCTTGGGTTGATCTTTACAACCACATCCCACGCAACTAT

GTGAACACTACTCTAAAATACACCGACCAGTTCAGGTAATTCTCTTAATACTTATTTCTT

CTTTTGATTTATGTTTCTGGCTTTTGTTTTTTTGCTGAAGTTTGTTGTGTTGTCAGGTTT

CTAGACAAAGCAGCGATTAAGTCACCCGAGGACAAGTTGAACTCTGATGGAGCGGCTTCA

GATCCATGGAAACTATGCACGTTGCAGCAAGTGGAAGAAGTGAAGTGCATTGTTAGAGTG

ATCCCAATCTGGCTTGCTTCCTCGATTTACTATCTTGCAATAAGTCTGCAAATGACTTAT

CCTGTCTTCCAAGCACTCCAGAGTGACCGGCGTTTGGGTTCTGGTGGCTTCAAGATTCCC

GGGGCAACCTATGTAGTGTTCTTAATGACGGGGATGACAATTTTCATCGTATTCTATGAC

CGTGTTCTTGTCCCGTCGCTCAGGAGAGTGACCGGGTTAGAAACTGGCATAACACTCTTA

CAAAGAATCGGAGCAGGGATTGTGTTTGCGATCTTGAGTTTGTTAGTGTCTGGATTTATA

GAGGAAAGGAGAAGAACCTTTGCGCTGACAAAACCAACACTCGGGATGGCGCCTAGGACA

GGAGAAATCTCTTCCATGTCAGCTATGTGGCTGATTCCGCAGCTCACACTTGCAGGCATA

GCAGAAGCTTTTGCTGCCATTGGACAGATGGAGTTTTACTACAAGCAGTTTCCTGAAAAC

ATGAGGAGCTTCGCCGGTTCTATCTTCTATATTGGTGGTGGGATTTCTAGCTACCTCGCT

AGCTTCTTGATCTCAACTGTTCATCGAACAACTGAGCATTCACCTACAGGGAATTGGTTA

GCTGAGGATCTGAACAAAGCCAAACTGGATTACTTCTATTTCATGCTCACAGGAATCATG

GTCGTTAACTTGGGTTACTTCTTGTTGATGGCTAAATGGTATAGATACAAAGGCAGTAAT

GATGAAGACATCACTGAGATTGAGACCAATGAAGAAGTGACCAAGAAACAGCAGCTACAA

GAAAAGGACTCTGTC**TGA**TCTGCAAACTTGTTTTATCTTTGGTCAAACGACCTCAGTTTC

TTCTTTCTCGTTGTACTTTTTTCTTTACTCCGAAGTCAATCCTGTTGTACTTTATATAGA

T

**CsGTR1-B (partial sequence)**

AGGAACATTATCGAACCTTCTTGTCTACCTAACTTCTGTATTCAACCTTAAGAGCTATAC

AGCTGCAACTATCATCAATGCCTTTAGTGGCACTATCAATTTCGGGACTTTCATCGCTGC

TTTCCTCTGTGACACTTACTTTGGTCGCTACAAGACTCTCTGTTTCGCTGTCATCGCTTG

TTTCATGGTACTTACATATATGTATATAATCTTTGATGACTTAACCTAGCGCCAATATTT

TTCTAAAACATTATCATCTTTGGTAGGGATCGTTTGTGATACTACTGACTGCTGCAATTC

CTTCATTGCACCCCGTTGCTTGCGGAAACAAAAGCACATGCGAAGGTCCAAGTGTGGGAC

AGATAATGTTCCTGCTCATGGGTTTAGGGTTTCTTGTGATAGGCGCTGGTGGTATCAGAC

CGTGTAATTTAGCGTTTGGTGCTGATCAGTTCAACCCCAAATCCGAATCAGGAAAGAAAG

GAATCAACAGTTTCTTCAACTGGTATTTCTTCACGTTCACGTTTGCGCAGATCGTCTCAC

TCACACTAATTGTGTATATCCAGTCAAATGTGAGTTGGACCATCGGTTTGACTATCCCTG

TGGTTCTTATGTTCTTGGCCTGCGTCATCTTCTTTGCTGGAGATAGATTGTATGTAAAAG

TGAAAGCCTCGGGTAGTCCTTTGGCTGGTATTGCTCATGTTATAGCAGCCGCGATCAAGA

AACGTGGTTTGAAGCCTGCTAAGCAGCCTTGGGTCGATCTTTACAACCACATCCCACGCA

ACTATGTAAACACTACTCTCAAATACACCGACCAGTTCAGGTAAATCTCTTTATACTTAT

TTGTTCTCTTGATTTATGTTTCTGGCTTTTGTTTTTTTGCTGAAGTTTGTTGCATGTGTT

GTCAGGTTTCTAGACAAAGCAGCGATTAAGACCCCGGAGGACAAGTTGAACTCTGATGGA

GCGGCTTCAGATCCATGGAAGCTATGCACGTTGCAGCAAGTGGAAGAAGTGAAGTGCATT

GTTAGAGTGATCCCAATCTGGCTTGCTTCTTCGATATACTATCTTGCAATAAGTATGCAA

ATGACTTATCCTGTCTTCCAAGCACTCCAGAGCGACCGGCGTTTGGGTTCTGGTGGCTTC

AGGATTCCGGGGGCAACCTATGTAGTGTTCTTAATGACGGGGATGACAATTTTCATCGTA

TTCTATGACCGTGTTCTTGTCCCGTCGCTCAGGAGAGTGACCGGGTTAGAAACTGGCATA

ACACTCTTACAAAGAATCGGAGCAGGGATTGTGTTTGCGATATTGAGTTTATTAGTGTCT

GGATTTATAGAGGAAAGGAGAAGAACCTTTGCGCTGACAAAACCAACACTTGGGATGGCG

CCTAGGACAGGAGAAATCTCTTCCATGTCAGCTATGTGGTTGATTCCGCAGCTAACACTT

GCTGGCATAGCAGAAGCTTTTGCTGCCATTGGACAAATGGAGTTTTACTACAAGCAGTTT

CCTGAAAACATGAGAAGCTTCGCCGGTTCTATCTTCTATATTGGTGGTGGGATTTCGAGT

TACCTCGCTAGCTTCTTGATCTCAACTGTTCATCGAACAACTGAGCATTCACCTACAGGA

AATTGGTTAGCTGAGGATCTGAACAAAGCCAAATTGGATTACTTCTATTTCATGCTCACG

GGAATCATGGTCGTTAACTTGGGTTACTTCTTGTTGATGGCTAAATGGTATAGATACAAA

GGCAGTAATGATGAAGACATCACTGAGATTGAGACCAGTGAAGAAGTGCCCAAGAAACAG

CAGCTACAAGACAAGGACTCTGTC**TGA**TATGGAAACTTGTTTTATCTTTTGGTCAACGAC

CTCAG

**CsGTR1-C**

**ATG**AAGAGCAGAGTCATCATCCTTAACCATAGAGACAAAAGAGATAAGAATTATAACAAC

AACAACAACAGCGACCTCTTCTGTAATTACACACAGATTGAGACTATGGAGAGAAAGCCC

CTTGAGGTTGAGACTACGACAACGAACCACCACTCCGCCGTCGATGATGTGACATCTACG

ATGGTCGATTCATTCGAGGAAGATCAGAAAAAACTTGTTTATAGAGGATGGAAAGTCATG

CCCTTCATCATTGGTAAACTCACCCTTTTGTTTTTCTCTTTCGGACATAAGTATATATTC

TTTTGTTGTGTTTTGTCGGAATTTTAGAAAAAAAAATGAAATATACTATAAAACATTTTG

TCCGTGATATTATTAAATTTTCTATTTTTTTTTTAACTTGTCTTAGACTTGACTTGTATC

CAAAAATCTCGACTTTGGAGATTTTGAGCGAAAAAAATGGTTAAAACAAAATTAAGATTT

GATTGAAACCCAGATCGAAGGAGATTTCGTACATTTTTTCTAGATTATAAAAAGATCTTT

ATGAAAAAAAGATATTTTAATTTTTTTTTTTAAATTAATTTTCCGTGGAAAAAATAGACT

TTGAATCAAGCAGTTAAGTTTCTGAAAAAAAGTATAGTAGATAAGAATTTTGAGATTTTT

CTGGATAAGACAAAAAATGATTAACTTCTGGGAGTTACAGAGCACTTAGAACCAAAAAAA

AAAAGAAAAAAGTCAGAACATCATCGAAAGTCCAATCTTTTGACTTGTTGTGTGTTTCGG

TTTCGAAGCTGTAACATTAAATGCAATAAATACTTTCTAACTTCATGAGAAGGTATTCTT

TATCATTGATTTATGTGGTGATATTGACAGGTAATGAGACATTTGAGAAGCTTGGGATCA

TAGGGACATTATCGAACCTTCTTGTCTACCTAACTTCTGTATTCAACCTTAAGAGTTATA

CAGCTGCAACTATCATCAATGCCTTTAGTGGCACTATCAATTTCGGGACTTTCATCGCTG

CTTTCCTCTGTGACACTTACTTTGGTCGCTACAAGACTCTCTGTGTCGCTGTCATCGCTT

GTTTCATGGTACTTATATTTATATAATCTTTTGATTAGTTAAGCCGGCGCCAATATTGTC

TAAACCATTTTCTATCTTTGTAGGGATCGTTTGTGATACTACTAACTGCTGCAATTCCTT

CATTGCACCCCGTTGCTTGCGGAAACAAAAGCACATGCGAAGGTCCAAGTGTGGGACAGA

TAATGTTCCTGCTCATGGGTTTAGGGTTTCTTGTGATAGGCGCTGGTGGTATCAGACCGT

GTAATTTAGCGTTTGGTGCTGATCAGTTCAACCCCAAATCCGAATCAGGAAAGAAAGGAA

TCAACAGTTTCTTCAACTGGTATTTCTTCACTTTCACGTTCGCTCAGATCATCTCACTCA

CGCTAATTGTGTACATCCAGTCAAATGTGAGTTGGACCATCGGTTTGACTATCCCTGTGG

TTCTTATGTTCTTGGCCTGCGTCATCTTCTTTGCTGGAGATAGACTGTATGTAAAAGTTA

AAGCCTCTGGTAGTCCTTTGGCTGGTATTGCTCATGTTATAGCAGCCGCTATCAAGAAAC

GAGGATTGAAGCCTGTTAAGCAGCCTTGGGTCGATCTTTACAACCACATCCCACGCAACT

ATGTAAACACTAGTCTCAAATACACCGACCAGTTCAGGTAAATCTCTTAATACTTATTTG

TTCTCTTGATTTATGTTTCTGGCTTTTGTTTTTTTTTGCTGAAGTTTGTTGTGTTGTCAG

GTTTCTAGACAAAGCAGCGATTAAGTCCCCCGAGGACAAGTTGAACTCAGATGGAGCGGC

TTCAGATCCATGGAAGCTATGCACGTTGCAGCAAGTGGAAGAAGTGAAGTGCATTGTTAG

AGTGATCCCAATCTGGCTTGCTTCTTCGATATACTATCTTGCAATAAGTATGCAAATGAC

TTACCCTGTCTTCCAAGCACTCCAGAGCGACCGGCGTTTGGGTTCTGGTGACTTCAGGAT

CCCCGGAGCAACCTATGTAGTGTTCTTAATGACGGGGATGACAATTTTCATCGTATTCTA

TGACCGTGTTCTTGTCCCGTCGCTCAGGAGAGTGACCGGGTTAGAAACTGGCATAACACT

CTTACAAAGAATCGGAGCAGGGATTGTGTTTGCGATATTGAGTTTATTAGTGTCTGGATT

TATAGAGGAACGGAGAAGAACCTTTGCGCTGACAAAACCGACACTCGGTATGGCGCCTCG

GACAGGAGAAATCTCTTCCATGTCAGCTATGTGGCTGATTCCGCAGCTCACACTTGCAGG

CATAGCAGAAGCTTTTGCTGCCATTGGACAAATGGAGTTTTACTACAAGCAGTTTCCTGA

AAATATGAGGAGCTTCGCCGGTTCTATCTTCTATATAGGTGGTGGGATTTCGAGCTACCT

CGCTAGCTTCTTGATCTCAACTGTTCATCGAACAACTGAGCATTCACCTACAGGGAATTG

GTTAGCTGAGGATCTGAACAAAGCCAAATTGGATTACTTCTACTTCATGCTCACAGGAAT

CATGGTCGTTAACTTGGGTTACTTCTTGTTGATGGCTAAATGGTATAGATACAAAGGCAG

TAATGATGAAGACATCACTGAGATTGAGACCAATGAAGAAGTGACCAAAAAACAGCAGCT

ACAAGAGAAGGACTCTGTC**TGA**TCTGCAAACTTGTTTTATCTTTGGTCAAACGACCTCAG

TTTCTTCTTTCTCGTTGTACTTTTTTCNNTACTCCGAAGTCA

**CsGTR2-A**

CTTCTTTTTAATTTGTTGTTGTAATAATTTACTTAGAGCAACAAA**ATG**GAGAGAAAACAT

CTTGAAGTTGAGTCCAAGGACCAAAACCCTTCCTCCGCCGTGTACGGTGGCTCTGCTACG

GCTGTTGACTCTGTTGATGAAGATGTTCAGAATCAGAAGAAACTCGTTTTTAGAGGCTGG

AAAGTCATGCCTTTTATTATTGGTAACACAAATATTTATGAATCATTTATCCATTTTTTT

CCTTCTTCTAATTTTAAACCTTTATTATTATTATTTTTGGTTCTATGGAGTTTTGTGATA

AATTCAGGAAACTGTTTTTCAAAAAAAAATCTATCCTTAAAAAAGAAAAGAAGAAGAAAA

ACATCAGTCTTGCTCGAAGACTTGTTAGTTTGATTTGTGACGAGGAGAGTTGTTTTTGTT

TTGTCAGATTCTCTGTGTTGTCTTAAACTTTGACTTAGTCAACGGTAGTTTAAGACCAAA

AAAAAAAGAGGAGATCGAGTTTTGTTTGAGATTTTATAGTAAACATATTATAATTCTTGA

TTCTTGGTTACAAATTAATTAAAAGACACAGATATCATATAATTCTTGATTCTTGGTTTG

GTTTCTGATTCAACCAAAAGCAATATTTAGAAAGTTTATCACAAACCTGTATGTTGGATC

AAAAAGTCTTACGCTATTGGTTCTTTGTTGTTGTTATAATCAGGAAATGAGACATTTGAG

AAGCTTGGGATCATTGGAACACTATCAAACCTTCTGGTTTATTTAACTGCAGTCTTCAAC

ATGAAGAGTATCACAGCTGCAACTATCATTAATGCCTTTAGTGGCACAATCAACTTTGGA

ACTTTCGTAGCTGCTTTCCTCTGTGACACTTACTTTGGTCGATACAAGACTCTAAGTGTC

GCGGTCATCGCCTGTTTTCTTGTAATGATCCTTCCTCTTTTTTTAATCTTTAGAAGGTTT

TAAAGTCATAAAAATCTGATCAGTGAATTGGGGTTTTTGTAGGGATCGTTTGTGATACTA

TTGACTGCAGCAGTGCCAAAACTGCATCCAGCTGCATGTGGAACAGCGGCAGATAGCGTG

TGTAACGGGCCAACTGGAGGCCAGATAGCGTTTCTACTGATGGGCCTCGGGTTCCTTGTT

GTTGGAGCAGGTGGGATCAGACCGTGTAATTTAGCTTTTGGTGCTGATCAGTTTAACCCG

AAGAGCGAATCAGGGAAAAGAGGGATTGACAGTTTCTTCAATTGGTACTTCTTTACCTTC

ACATTCGCGCAGATCTTGTCGCTGACTCTAGTCGTGTACATTCAGTCTAATGTAAGTTGG

ACGATCGGTTTAACCATCCCGGCCGTTCTAATGTTCTTGGCCTGCTTGATTTTCTTTGCG

GGAGATAAGTTGTATGTAAAAATCAAAGCCTCTGGTAGTCCATTGGCCGGTATAGCTCAA

GTTATAGCGGTTGCGATCAAGAAACGTGGATTAAAGCCCGTGCAACAGCCTTGGCTTAAC

CTTTACAATTACTACCCACCAAAATACGCAAACTCCAAGCTTAAATACACCGACCAATTC

AGGTAAAAACACTCACACACACCAAAATATATTGTCACTTTCTTTATATGTTTTATGTTT

ACTGAAAATGCTTTCTTGTATTTGGCAGATTTCTAGATAAAGCGGCCATCATGACTCCTA

AAGACAAGTTGGAGTTAGATGGTAAGCCTGCTGATCCGTGGATGCTCTGTACAATGCAAC

AAGTTGAAGAAGTGAAGTGCATTGTTAGAGTGCTTCCTATATGGTTTGCTTCATCAATCT

ACTACTTGACCATAACTCAACAAATGACTTATCCCGTCTTCCAAGCCCTTCAGAGCGACC

GTCGCTTAGGATCAGGAGGATTTGTGATTCCTGCTGCCACCTACGTTGTTTTCTTGATGA

CCGGAATGACAATCTTCATCGTAGTCTACGACCGTGTGCTCGTGCCTACCATGAGAAGAA

TCACTGGTCTAGACACAGGGATAACGCTCTTGCAGAGAATCGGAACTGGCATTTTCTTCG

CTACCGCAAGCTTAATAGTCTCAGGATTTGTCGAGGAACGAAGGAGAACATTCGCTCTGA

CTAAACCTACACTTGGTATGGCGCCACGAAGAGGAGAGATCTCCTCAATGTCAGCTATGT

GGCTGGTCCCGCAGCTCTCACTGGCGGGTATAGCCGAGGCATTTGCAGCGATTGGACAAA

TGGAGTTTTACTACAAGCAGTTCCCTGAAAACATGAGGAGTTTTGCAGGATCCATATTTT

ACGTAGGAGGAGGGGTTTCGAGTTACCTTGGTAGCTTCTTGATTGCAACAGTTCACAGGA

CGACGCAGACCTCGTCCGGGGGTAACTGGTTAGCTGAGGATCTGAACAAAGGAAGATTGG

ATCTCTTCTATTTCATGATAGCCGGAATGTTGGCAGTAAACTTCGTTTATTTCTTGGTGA

TGTCAAGATGGTATAGGTACAAAGGAAGTGATGATGAAGTGACAACATATGAAACCAATG

AAGATATCATCAAACAGCAGGATAAGAACGTTGCC**TGA**TGATTATGTGTTTCTCTCATCT

TCTGG

**CsGTR2-B**

GATCTATTTTCTTCTTTTTTGTTGTTGTAATAATTTACTTGTAGCAACAAA**ATG**GAGAGA

AAACATCTTGAAGTTGAGTCCAAGGACCAAAACCCTTCCTCCGCCGTGTACGGTGGCTCT

GCTACGGCTGTTGACTCTGCTGATGAAGATGTTCAGAATCAGAAGAAAGTCGTTTTTAGA

GGCTGGAAAGTCATGCCTTTTATTATTGGTAACACAAATATTATGAATCATTTATCCATT

TTTTTCCTTCTTCTAATTTAAAACCTTTATTATTATTTTTTGGTTCTATGGAGTTTTGTG

ATAAATTCTGGAAACTGTTTAAAACAAAAAAATCTATACTAAAAAAAGAAAAGAAGAAAA

ACATCAGTCTTGCTCGAAGTCTTCGAAGACTTGTTAGTTTGATTTGTGACGAGGAGAGTT

GTTTTTGTTTTGTCTTGGTTTTGTCAGATTCTCTGTGTTATCTTAAACTTTGACTTAGTC

AACGGTAATTTAAGACAAAAACAAGAGGGAGAGAGAGATGTCGAGATTTTATAACATATA

TTTGGTAAAAAAAATTAACTAAAACACACAGATTTATATAATTCTTGATTCTTAATTTGG

TTTCTGATTCAACCAAAAGCAATAGTTTGAAAGTTATATCACAAACCTCTATGTTTGATC

AAAGTCTTACGCTATTGGTTCTTTGTTGTTGTTATAATCAGGAAATGAGACATTTGAGAA

GCTTGGGATCATTGGAACACTATCAAACCTTCTGGTTTATTTAACTTCAGTCTTCAACAT

GAAGAGTATCACAGCTGCAACAATCATTAATGCCTTTAGTGGCACAATCAACTTCGGAAC

TTTCGTAGCTGCTTTCCTCTGTGACACTTACTTTGGTCGATACAAGACTCTTAGTGTCGC

GGTCATCGCCTGTTTTCTTGTACTGATCCTTCCTCTTTTTTTAATCTTTAGAAGGTTTTA

AAGTCATAAAAATCTGATCAGTGAATTGGGGTTTTTGTAGGGATCGTTTGTGATACTAAT

GACTGCTGCAGTGCCAAAATTGCATCCAGCTGCATGTGGAACAGCAGTAGATAGCGTGTG

TAACGGGCCAACTGGAGGCCAGATAGCGTTTCTACTGATGGGCCTCGGGTTCCTTGTTGT

GGGAGCAGGTGGGATCAGACCGTGTAATTTAGCTTTTGGTGCTGATCAGTTCAACCCGAA

GAGCGAATCAGGGAAAAGAGGGATTGACAGTTTCTTCAATTGGTACTTCTTTACCTTCAC

ATTCGCGCAGATCTTGTCGCTGACTCTAGTCGTGTACATTCAGTCTAATGTAAGTTGGAC

GATCGGTTTAACCATCCCGGCCGTTCTAATGTTCTTGGCCTGCTTGATTTTCTTTGCGGG

CGATAAGTTGTATGTAAAAATCAAAGCCTCTGGTAGTCCATTGGCCGGTATAGCTCAAGT

TATAGCGGTTGCGATCAAGAAACGTGGATTAAAGCCCGTGCAACAGCCTTGGCTTAACCT

TTACAATTACTACCCACCAAAATACGCAAACTCCAAGCTTAAATACACCGACCAATTCAG

GTAATAACACACACACACACCAAAAAATATTGTCACTTTCTTGATATGTTTTATGTTTAC

TAAAAATGCTTTCTTGTATTTGGCAGATTTCTAGATAAAGCGGCCATCATGACTCCTAAA

GACAAGTTGGAGTTAGATGGTAAGCCTGCTGACCCGTGGATGCTCTGTACAATGCAACAA

GTTGAAGAAGTGAAGTGCATTGTTAGAGTGCTTCCTATATGGTTTGCTTCATCAATCTAC

TACTTGACCATAACTCAACAAATGACTTATCCCGTCTTCCAAGCCCTCCAGAGCGACCGT

CGCTTAGGATCAGGAGGATTTGTGATCCCTGCAGCCACCTACGTGGTTTTCTTGATGACA

GGAATGACAATCTTCATCGTAGTCTACGACCGTGTGCTCGTACCTACCATGAGAAGAATC

ACTGGTCTAGACACAGGGATAACGCTCTTGCAGAGAATCGGAACTGGCATTTTCTTCGCT

ACCGCAAGCTTAATAGTCTCAGGATTTGTCGAGGAACGAAGGAGAACGTTCGCTCTGACT

AAACCAACACTTGGTATGGCGCCACGAAGAGGAGAAATCTCTTCAATGTCAGCTATGTGG

CTGATTCCGCAGCTCTCACTGGCGGGTGTAGCCGAGGCATTTGCAGCGATTGGACAAATG

GAGTTTTACTACAAGCAGTTTCCTGAAAACATGAGGAGTTTCGCAGGATCCATATTTTAC

GTAGGAGGAGGGGTTTCGAGTTACCTTGGTAGCTTCTTGATTGCAACAGTTCACAGGACG

ACGCAGACCTCGTCCGGGGGTAACTGGTTAGCTGAGGATTTGAACAAAGGAAGATTGGAT

CTCTTCTATTTCATGATCGCCGGAATGTTGGCAGTAAACTTCGTTTATTTCTTGGTGATG

TCAAGATGGTATAGGTACAAAGGAAGTGATGATGAAGTGACAACATATGAAACCAATGAA

GATATCATCAAACAGCAGGACAAGAACGTTGCC**TGA**TGATTATGTGTTTCTCTCNTCTTC

**CsGTR2-C**

**ATG**GAGAGAAAACATCTTGAAGTTGAGTCCAAGGACCAAAACTCTTCCTCCGCCGCTTAC

GGTGGCTCTGCTACGGCTGTTGACTCTGTTGATGAAGATGTTCAGAATCAGAAGAAAGTC

GTTTTTAGAGGCTGGAAAGTCATGCCTTTTATTATTGGTAACACAACAAATCTTTATGAT

CATTTATCCATTTTTAACTTTATTATTATTTTGGTTCTATGGAGTTTTGTGATAAATTCA

GGAAACTGTTAAAAAAAAAAAAATCTATATAAAAAAAGAAAAGAAGAAAAACATCAGTCT

TGCTCGAAGTCTTCGAAGACTTGTTAGTTTGATTTGTGACGAGGAGAGTTGTTTTTGTTT

TGTTTTGGTTTTGTCAGATTCTCTGTGTTGTCTTAAACTTTGACTTAGTCAACGGTAATT

TAAGACAAAAAAAAAAAAGAGGGAGATCGAGTTTTGTTTGAGATTTTATACTAACACATA

GTATTTGGTCAAAAACCAATTAAAACACACAGATTTATATAATTCTTGATTCTTAATTTG

GTTCCTAATTCAACCAAAAGCAATATGTTGAAAGTTTATCACAAACCTCTATGTTTGATC

AAAGTCTTACGCTATTGGTTCTTTGTTGTTGTTATAATCAGGAAATGAGACATTTGAGAA

GCTTGGGATCATTGGAACACTATCAAACCTTCTGATTTATTTAACTGCAGTCTTCAACAT

GAAGAGTATCACAGCTGCAACAATCATTAATGCCTTCAGTGGCACAATCAACTTCGGAAC

TTTCGTAGCTGCTTTCCTCTGTGACACTTACTTTGGTCGATACAAGACTCTTAGTGTCGC

CGTCATCGCCTGTTTTCTTGTACTGATCCTTCCTCTTATTTTTAAATTCTTTAGAAGGTT

TTAAAGTCATAAAAAATCTGATCAGTGAATATGGTTTTTGTAGGGATCGTTTGTGATACT

ATTGACTGCTGCAGTGCCAAAACTACATCCAGCTGCATGTGGAACAGCGGCAGATAGCGT

GTGTAACGGGCCAACTGGAGGCCAGATAGCGTTTCTACTGATGGGCCTCGGGTTCCTTGT

TGTTGGAGCAGGTGGGATCAGACCGTGTAATTTAGCTTTTGGTGCTGATCAGTTTAACCC

GAAAAGCGAATCAGGGAAAAGAGGGATTGACAGTTTCTTCAATTGGTACTTCTTTACCTT

CACATTCGCGCAGATCTTGTCGCTGACTCTAGTCGTGTACATTCAATCTAATGTAAGTTG

GACGATCGGTTTAACCATCCCGGCCGTTCTTATGTTCTTGGCCTGCTTGATTTTCTTTGC

GGGCGATAAGTTGTATGTAAAAATCAAAGCCTCGGGTAGTCCATTGGCCGGTATAGCTCA

AGTTATAGCGGTTGCGATCAAGAAACGTGGATTAAAGCCTGTGCAACAGCCTTGGCTTAA

CCTTTACAATTACTACCCTCCAAAATACGCAAACTCCAAGCTTAAATACACCGACCAATT

CAGGTAATAACACTCACACAGACACCACAAAACTAGTCTTTTCACTTTCTTGATATGTTT

TAGGTTTACTGAAAATGCTTCCTTGTATTTGGCAGATTTCTAGACAAAGCGGCCATCATG

ACTCCTAAAGACAAGTTGGAGCTAGATGGTAAGCCTGCTGATCCGTGGATGCTATGTACA

ATGCAACAAGTTGAAGAAGTGAAGTGCATTGTGAGAGTGCTTCCTATATGGTTTGCTTCA

TCAATCTACTACTTGACCATAACTCAACAGATGACTTACCCCGTCTTCCAAGCCCTTCAG

AGCGACCGTCGCTTAGGATCAGGAGGATTTGTGATTCCTGCAGCCACCTACGTTGTTTTC

TTGATGACAGGAATGACAATCTTCATCGTAGTCTACGACCGTGTACTCGTGCCTACCATG

AGAAGAATCACTGGTCTAGACACCGGGATAACGCTCTTGCAGAGGATCGGAACTGGCATT

TTCTTCGCTACCGCAAGCTTAATAGTCTCAGGATTTGTCGAGGAACGAAGGAGAACGTAT

GCTCTGACTAAACCTACACTTGGTATGGCGCCAAGAAGAGGAGAAATCTCTTCAATGTCA

GCTATGTGGCTGATTCCGCAGCTCTCACTGGCGGGTGTAGCCGAGGCATTTGCAGCGATT

GGACAAATGGAGTTTTACTACAAGCAGTTTCCTGAAAACATGAGGAGTTTTGCAGGATCC

ATATTTTACGTAGGAGGCGGAGTTTCGAGTTACCTTGGTAGCTTCTTGATTGCAACAGTT

CACAGGACGACGCAGACCTCGTCCGGGGGTAACTGGTTAGCTGAGGATTTGAACAAAGGA

AGATTGGATCTCTTCTATTTCATGATCGCTGGAATGTTGGCAGTAAATTTCGTTTATTTC

TTGGTGATGTCAAGATGGTATAGGTACAAAGGAAGTGATGATGCAGTGACAACATATGAA

ACCAATGAAGATATCATCAAACAGCAGGACAAGAACGTTGCC**TGA**TGATTAT

**CsMYB28-A**

TCTTATCAAGTTAAACAGTATCCTCTGAAATTTTAATTTTCAAAAGATTTATATATTTCT

CTCTATTTTTATATCTTGAGTGTTTGTGAGAGATTATATGAAATTTACCCATTATTGGTC

ATATATATATCGGAAAAAGA**ATG**TCAAGAAAGCCATGTTGTGTCGGAGAAGGGCTCAAGA

AAGGGGCGTGGACCACCGAGGAGGATAAGAAACTCATCTCCTACATCCACGACCACGGCG

AAGGAGGCTGGCGCGACATTCCCCAAAAAGCTGGTTTGTACATGCACACACATCTATACA

TGATTAAATAGTTTTTTTTTATTACCAAAAAATAATAATGATGAAGATGGTGTTTGATTA

GGGTTGAAACGGTGTGGAAAGAGCTGTAGACTGCGATGGACCAATTACCTTAAACCTGAG

ATCAAACGAGGCGAGTTTAGTTCAGAGGAAGAGCAGATTATCATCATGCTTCATGCTTCT

CGAGGCAACAAGTACGTTTCTATGTTTATATATATAACAAACAAACAAAAAATGTGTGCG

CGTGTATCGATCCGATGGAATGTATAATGAAATGCATGAAATAAATTTATATAAGATTTA

CTTTAATTTGTAGACATGATTATCATCTTAATTCTGTCTTCGGTCTATCTATTCATCTTC

TTTCCTCTAGGTGGTCGGTCATAGCGAGACATTTACCTAGAAGAACGGACAACGAGATCA

AGAACTATTGGAACACCCATCTCAAAAAACGTTTGGTTGAGCAGGGTATTGATCCCGTGA

CACACAAGCCACTAGCTTCTAATTCCAACCCAACGGTTCATGAGAATTTGAATTCCCTAA

ATGCCTCCAGTCCCGACAACCAATACTCACGGTCGAGCTCAATGCCTTCTCTGTCTCCAC

CTCTATCCGGTTGCAACATGGTTTCCGAGGCGACTGAGTTAAGCAGCAATGATGGAACAC

CAATGCAAGGCGGCGGTTCCTTGAGTTGCAAGAAAAATTTCAAGAAATCAAGTTCTACAT

CAAGGCTATTGAACAAAGTTGCAGCTAAGGCCACTTCCATTAAGGATATATTGTCGGCTT

CCATGGAAGGTAGCTTAAGTGCTACTACAATATCACATGCAAGCTTTTTCAATGGCTTCT

CTGAACAGATCTGCAGTGAAGAGGATAGTTCTAATGCATCCCTGACAAATCTCGCTGAAT

TTGATCCCTTCTCCCAATCATCATTGTACCCTGAGCATGAGATCAATGCTACTTCTGATC

TGGACATGGGCCAGGATTATGATTTCTCACATTTTCTCGAAAAACTTGGGGTTGGTAACC

ACGATGAGGAGAATAATATGAATGTCGAGTACAACCATGATCTCCTTATGTCTGATGTTT

CCCAAGAAGTCTCATCAACTAGCGTTGACGATGAAGACAACATGGTAGGAAACTTCGAGG

GGTGGTCAGATTATCTTCTTGACCATGCCAATTTTATGTACGACACCGACTCAGATTCCC

TCGAAAAGCATTTCATA**TGA**CTCTTCCTATCCAAGCAAAAAGGTTTCAAACTATCTGAAG

ACGTATATCAATGGTTCTATATCGATCCGAGGCTTACTATAACTCGCTCCATGGAGATGT

TGTGTATGTATCTAGTATTTGGGTTGGTCCAAGGTCGTCTCATGGTTAATAATCCCAAGT

TAGTAGTACGGTGCTAAATAAGGGATATATATCCGCAAGAC

**CsMYB28-B**

CTTATCTAGTTGAACAATATCCTCTGAAATTTTAATTTTCAAAATATTTATATATTTCTC

TATTTTTAAATATTGAGTGTTTGTGAGAGATTAT**ATG**AAATTTACCCATTATTGGTCATA

TATATATCGGAAAAAAATGTCAAGAAAGCCATGTTGTGTCGGAGAAGGGCTCAAGAAAGG

GGCGTGGACCACCGAGGAGGATAAGAAACTCATATCCTACATCCACGACCACGGTGAAGG

AGGCTGGCGCGACATTCCCCAAAAAGCTGGTTTGTACATGCACACACATCTATACATGAT

TAAATAGTTTTTTTTTTAATTTAAAAAACAAATAATGATGAAGATGGTGTTTGATTAGGC

TTGAAACGGTGTGGAAAGAGCTGTAGACTGCGATGGACCAATTACCTTAAACCTGAGATC

AAACGAGGCGAGTTTAGTTCAGAAGAAGAGCAGATTATCATCATGCTTCATGCTTCTCGA

GGCAACAAGTATGTTTCTATGTTTATATATATAACAAAAAAAAAATGTGTGCGCGTGTAT

CGATCCGATGGAATGTATAGTGAAATGCATGAAATAAATTTGTATAATATTTAGTTTAAA

TTGAAGACATGATCATCATCTTAATTCTGTCTTCGGTTTATCTATTCATCTTCTTTCCTC

TAGGTGGTCGGTCATAGCGAGACATTTACCTAGAAGAACGGACAACGAGATCAAGAACTA

TTGGAACACCCATCTCAAAAANCGTTTGGTTGAGCAGGGTATTGATCCCGTGACTCACAA

GCCACTAGCTTCTAATTCCAACCCAACGGTTCATGAGAATTTGAATACCCTAAATGCCTC

TAGTCCCGACAACCAATACTCACGGTCGAGCTCAATGCCTTCTCTGTCTCCACCTCTATC

CGGTTGCAACATGGTTTCCGAGGTGACTGAGTTAAGCAGCAATGATGGAACACCAATTCA

AGGCGGCGGTTCCTTGAGTTGCAAGAAAAATTTCAAGAAATCGAGTTCTACATCAAGGCT

ATTGAACAAAGTTGCAGCTAAGGCCACTTCCATTAAGGATATTTTGTCGGCTTCCGTGGA

AGGTAGCTTAAGTGCTACTACAATATCACATGCAAGCTTTTTCAATGGCTTCTCTGAACA

GATCCGCAGTGAAGAGGATAGTTCTAATGCATCCCTGACAAATCTCGCTGAATTTGATCC

CTTCTCCCAATCATCATTGTACCCTGAGCATGAGATCAATGCTACTTCTGATCTGGACAT

GGGCCAGGATTATGATTTCTCACATTTTCTCGAAAAACTTGGGGTTGGTAACCACGATGA

GGAGAACAATATGAATGTCGAGTACAACCATGATATCCTTATGTCCGATGTTTCCCAAGA

AGTCTCATCAACTAGCGTTGACGATCAAGACAACATGGTAGGAAACTTCGAGGGGTGGTC

AAATTATCTTCTTGACCATGCCAATTTTATGTATGACACCGACTCAGATTCCCTCGAAAA

GCATTTCATA**TGA**CTCTTCCTATCCAAGCAAAAAGGTTTCAAACTATCTGAAGACGTATA

TCAATGGTTCTATATCGATCCGAGGCTTACTATAACTCGCTCCATGGAGGTGTTGTGTAT

GTATCTAGTAT

**CsMYB28-C**

ATTCTTTCTTATCAAGTTAAATTCCTCTGAAGTTTTACTTTTCAAAAATATTTATATATT

TCTCTATATTTTTATATATTGAGTGTTTGTGAGAGACTATATGAAATTTACCCATTATTT

GGTCATATATATATCGGAAAAAAA**ATG**TCAAGAAAGCCATGTTGTGTCGGAGAAGGGCTC

AAGAAAGGGGCGTGGACCACCGAGGAGGATAAGAAACTCATATCCTACATCCACGACCAC

GGTGAAGGAGGCTGGCGTGACATTCCCCAAAAAGCTGGTTTGTACATGCACACACATCTA

TACATGATTCAATAGTTTTTTTTTTTTTTTTTTTTTAATAATAATAATGATGAAGATGGT

GTTTGATTAGGGTTGAAACGGTGTGGAAAGAGCTGTAGACTGCGATGGACCAATTACCTT

AAACCTGAGATCAAACGAGGCGAGTTTAGTTCAGAGGAAGAGCAGATTATCATCATGCTT

CATGCTTCTCGAGGCAACAAGTACGTTTCTATGTTTATATATATAACAAACAAACAAAAA

ATATGTGTGCGCGTGTATCGATCCGATGGAATGTATAGTGAAATGCATGAAATAAATTTG

TATAATATTTACTTTAATTTGAAGACATGATCATCATCTTAATTCTGTCTACGGTCTATC

TATTCATCTACTTTCCTCTAGGTGGTCGGTCATAGCTAGACATTTACCTAGAAGAACGGA

CAACGAGATCAAGAACTATTGGAACACCCATCTCAAAAAACGGTTGGTTGAGCAGGGTAT

TGATCCCGTGACTCACAAGCCACTAGCTTCTAATTCCAACCCAACGGTTCATGAGAATTT

GAATTCCCTAAATGCCTCTAGTCCCGACAACCAATACTCACGGTCGAGCTCAATGCCTTC

TCTGTCTCTACCTCTATCCGGTTGCAACATAGTTTCCGAGGTGACTGAGTTAAGCAGCAA

TGATGGAACACCAATGCAAGGCGGCGGTACCTTGAGATGCAAGAAAATTTTCAATAAATC

GAGTTCTACATCAAGGCTTTTGAACAAAGTTGCAGCTAAGGCCACTTCCATCAAAGATAT

ATTGTCGGCTTCCATGGAAGGTAGCTTAAGTGCTACTACAATATCACATGCAAGCTTTTT

CAATGGCTTCTCTGAGCAAATTCGCAGTGAAGACGATAGTTCTAATGCATCCCTGACAAA

TCTCGCTGAATTTGATCCCTTCTCCCAATCATCATTGTACCCTGAGCATGACATCAATGC

TACTTCTGATCTCGACATGGGCCAGGATTATGATTTCTCACATTTTCTCGAAAAACTTGG

GGTTGGTAACCATGACGAGGAGAACAATATGAATGTCGAGTACAACCATGATCTCCTTAT

GTCTGATGTTTCCCAAGAAGTCTCATCAACTAGCGTTGACGATCAAGACAACATGGTAGG

AAACTTCGAGGGGTGGTCAAATTATCTTCTTGACCATGCCAATTTTATGTATGACACCGA

CTCAGATTCCCTCGAAAAGCATTTCATA**TGA**ATCTTCCTATCCAAGCAAAAAGGTTACAA

ACTATTTGAAGACGTATATCAATGGTTCTGTATCGATACGAGGCTTGTCATTTACTATAA

CTCGC

**CsMYB29-A**

CTTATTTTCTTGTTTCTTCATCTTTAATTAGGTTGTATGTAAAATCAATATCATAAAAGAAAGAAAAAAA

**ATG**TCAAGAAAGCCATGTTGTGTGGGAGAAGGGCTGAAGAAAGGAGCATGGACCGCCGAAGAAGACAAGA

AACTCATCTCTTACATTCATGAGCACGGTGAAGGAGGCTGGCGTGATATTCCCCAAAAAGCTGGTACATT

TGTACTTAATTATATTATGTTTATATACTACTCCTTCTATTTCATAAAAATTGATGTTTTAAGATATTTT

TTTGTCCCATAAACATTGATGTTTTGTAAACTTCAAAAAGTAATCATTGAAAATATTTAAAATTTTGAAT

TGTTATTGGTTTAAAATTAAATAATTTCTCTTTAGTCAAATAAAAAAATATATTTAAACTCAGTAATCAT

TGTTTTCTTAAAATGTGTAAAACATTTTAAACAATCAATTTTTTTTAGTTTGTTGTTTTAGCGAAGGCTG

GTGTTTTGCATTTCCTATGCATTTTTTATTTTTTTTATTTTTGGTGAAATTCCTATGCATTTCCAATTAG

TTGATACCAATACGTCAAATGAACGAATATAAAAAACCTAATGAATATGAAAATCAAGTAATATCAAATT

CAATAAAAATGATTAATTACATACAATAGAACTAATTATAGAATCTTATTAATTAGTAGATAATAAATTT

TTATGAAAGAATTTTTTTGTATATATATATATATATTGTCTAACAGTTGGTTTTATAATCATGACAATGG

TGGTTAATTAGGACTCAAACGATGTGGAAAGAGTTGTAGACTGCGATGGGCTAACTATTTGAAACCTGAT

ATCAAGAGAGGAGAGTTTAGCTACGAGGAGGAACAGATTATCATCATGCTTCACGCTTCTCGCGGCAACA

AGTAATTAAAAATGCTTCTAGCTTGTCAAAATCCACACAAATAAGTGTAATTTAGCACATAGATTTTAAA

GTTGGATTTATTTTTCTTAATAGGTGGTCGGTCATAGCTAGACATTTACCCAAAAGAACGGACAACGAGA

TCAAGAACTATTGGAACACGCATCTTAAAAAACGTCTGATCGATCAAGGAATCGATCCCGTCACCCACAA

GCCACTTGCCTCTAATCCTAATCCAGGCACGCCCAAGACTTCTGATCCCAACGATGATGAACAATCACAA

TCAGGTTCGATGTCTCCAAAGTCTCTTCCTCCTTCTTCTAACTCCTACAATCTACAAGAGACAAGCAGCA

GTGATGAGACACCGAGACACGGAGCTTCCTTGACCTCCAAGAAACACTTGTTTAAAAGGTCAAGTTCTAC

ATCAAAAGTGTTAAACAAAGTTGCAGCTAGGGCTGCTTCCATTGGAAATATTTTATCTGCCTCCATGGAA

GGAACCTTGATCAGCTCTACATCCTTGTCTCCTCCATGTCTCAATGATGACTTTTCCGAAACTAGTCAAC

TTCAGATTGACGAGTATGATCCATTCTCTCAGCCATCCGAACACATAATTCCCGATGATATCAGCATGAA

CACGGAACTCAACAATTCCGCATACGATTTCTCGCAGTTTCTTGAGCAGTTCGGTAACAACGAAGGCGAC

AACATTCAAGATCTACTTATGTCTGATGTCTCATCACCAAGCGTTGATGAATGCGATATTATGCAAAACA

TAACCGATTGGTCAAACTATCTTGTTGAAGATTCCGATTTTAATCATGACACGAACCAAGATTGCGACGA

CAGGAACTTCATA**TAA**TCATATAATCCATGGTTCTCGTAGATATTTGTCAAGTTATTGTCAGTTTCACTG

TAGAAGTTGCTATATTCAATAAAGATTCTCAGTCTGAGAAACTTGTGATCCCGTGGATCAATTAGTATTT

GATTTGCGGGAGACACGAGTTTTTTTTTT

**CsMYB29-B**

TTTCTTGTTTCTTCATCATTGATTAGGTTGTATGTAAAATCTATAATCATAAAGAAAGAAAAAA**ATG**TCA

AGAAAGCCATGTTGTGTGGGAGAAGGGCTGAAGAAAGGAGCATGGACCGCCGAAGAAGACAAGAAACTCA

TCTCTTACATTCATGAGCACGGTGAAGGAGGCTGGCGTGATATTCCCCAAAAAGCTGGTATATTTCTACT

TAATTATATTATGTTATTTTTTTTTAACAAAGTACTTCATTATATTATGTTTATATAGTTGTTTATATAC

CACCAGTCCACCACCTTATTTTTTTTTTAGTTTTTGGTTTAGTGAAGACTGGTGTTTTGCATTTCCTATG

CATTTTCAATTAGTTGATATCAATACGTCAAATGCACGAATATTTGAAACCTAATTGATATGAAAATCAA

GTAATATCAAATTCAATAAAAATGATTAATTAATTAAATACAATATAATTATAGAATCTTTTTTTTTAAC

AACGAATCTTATGAATTAGTAGAGAATAAATCTTTATGAAAGAAAATAAATTTTGTATATGTATATACTG

TCTAACAGTTGGTTTTATAATCATGACAATGGTGGTATAATTAGGACTCAAACGATGTGGAAAGAGTTGT

AGACTGCGATGGGCTAACTATTTGAAACCTGATATCAAGAGAGGAGAGTTTAGCTACGAGGAGGAACAGA

TTATCATCATGCTTCACGCTTCTCGCGGCAACAAGTAAAAATGCTTCTAGCTTGTCAAACTCCACACAAT

AAGTGTAATTTAGCACATATATTAAAGTTTGGATATTATTCTTCTTAATAGGTGGTCGGTCATAGCGAGA

CATTTACCCAAAAGAACAGACAACGAGATCAAGAACTATTGGAACACGCATCTTAAAAAACGTCTTACCG

ATCAGGGAATCGATCCCGTCACCCACAAGCCACTTGCCTCTAACCCTAATCCAGGCACGCCCAAGACTTC

TGATCCCAACGATGATGAACAATCGCAATCAGGTTCGATGTCTCCAAAGTCTCTTCCTCCTTCTTCAAAC

TCCAACAATCTACATCATGAGACAAGCAGCAGTGATGAGACACCGAGACACGGTGCTTCCTTGACCTCCA

AGAAACACTTGTTTAAGAGGTCAAGTTCTACATCAAAAGTGTTAAACAAAGTTGCAGCTAGGGCTGCTTC

CATTGGAAATATTTTATCAGCCTCCATGGAAGGAACCTTGATCAGCTCTACATCCTTGTCTCCTCCATGT

CTCAATGATGACTTTTCCGATACTAGTCAACTTCAGATGGACGAGTATGATCCATTCTCTCTGCCATCCG

AACACATTATTCCCGATGATATCAGCATGAACACTGAACTCAACAATTCCGCATACGATTTCTCGCAGTT

TCTTGAGCAGTTTGGTAACAACGAAGGCGACAACATTCAAGATCTACTTATGTCTGATGTCTCATCACCA

AGCGTTGATGAATGCGATATTATGCAGAACATAACCGAGTGGTCAAATTATCTTGTTGAAGATTCCGATT

TTAATCGTGACACGAACCAAGATTGCGACGACATGAACTTCATA**TAA**TCCATTGATTGCTTACCGGAACA

GAGTTCACCGGTACTTTGATGGTTCTCGTAGATATTTGTCAAGTTATTGTCAGGTTCACAGTAGAAGTTG

CTATATTCAATAAAAGA

**CsMYB29-C**

TTTCTTCATCTTTAATTAGGTTGTATGTAAAATCAATATCATAAAAGAAAGAAAAAAA**ATG**TCAAGAAAG

CCATGTTGTGTGGGAGAAGGGCTGAAGAAAGGAGCATGGACCGCCGAAGAAGACAAGAAACTCATCTCTT

ACATTCATGAGCACGGTGAAGGAGGCTGGCGTGATATTCCCCAAAAAGCTGGTACATTTGTACTTAATTA

TACTCCTATGTTTATATACTTTTTAATATACGAACCTTAATTTTTTTTAGTTTTTGGTTTGGTGAAGGCT

GGTGTTTTGCATTTCCTATGCATTTCCAATTAGTTGATATCAATACGTCATATGAACGAATACTTGAAAC

CTAATTAATATGAAAATCAAGTAATATCAAATTCAATAAAAATGATTATAATCAATATAACTAATTATAG

AATCTTATTAATTAATTAGTAGATAATCATTTTTTATGAATTTTTTTTGTTTTGTAGATGTATATATTGT

CTAACAGTTGGTTTTATAATCATGACAATGGTGGTTAATTAGGACTCAAACGATGTGGAAAGAGTTGTAG

ACTGCGATGGGCTAACTATTTGAAACCTGATATCAAGAGAGGAGAGTTTAGCTACGAGGAGGAACAGATT

ATCATCATGCTTCACGCTTCTCGCGGCAACAAGTAATTAAAAATGCTTCTAGCTTGTCAAAATCCACACA

AATAAGTGTAATTTAGCACATATATTAAAGTTTGGATATAATTTTTACTCTTCTTAATAGGTGGTCGGTC

ATAGCAAGACATTTACCCAAAAGAACGGACAACGAGATCAAGAACTATTGGAACACGCATCTTAAAAAAC

GTCTGGTCGATCAGGGAATCGATCCCGTCACCCACAAGCCACTTGCCTCTAACCCTAATCCAGGCACGCC

CAAGACTTCTGATCCCAACGATGATGAACAATCGCAATCAGGTTCGATGTCTCCAAAGTCTCTTCCTCCT

TCTTCAAACTCCTACAATCTACAAGAGACAAGCAGCAGTGATGAGACACCGAGACACGGTGCTTCCTTGA

CCTCCAAGAAACACTTGTTTAAGAGGTATTCTACATCAAAAGTGTTAAACAAAGTTGCAGCTAGGGCTGC

TTCCATTGGAAATATTTTATCAGCCTCCATGGAAGGAACCTTGATCAGCTCTACATCCTTGTCTCCTCCA

TGTCTCAATGATGACTTTTCCGATACTAGTCAACTTCAGATGGACGAGTATGATCCATTCTCTCAGCCAT

CCGAACACATAATTCCCGATGATATCAGCATGAACACGGAACTCAACAATTCCGCATACGATTTCTCGCA

GTTTATTGAGCAGTTCGGTAACAACGAAGGCGACAACATTCAAGATCTGCTTATGTCTGATGTCTCATCA

CCAAGCGTTGATGAATGCGATATTATGCAAAACATAACCGATTGGTCAAATTATCTTGTTGAAGATTCCG

ATTTTAATCATGAAACGAACCAAGATTGCGACGACAGGAACTTCATA**TAA**TCCATTGATTGCTTACCGGA

ACAGAGTTCACCAGTACTTTGATGGTTCTCGTAGATATTTGTCAAGTTATTGTCAGGTTCACAGTAGAAG

TTGCTATATTCAATAAAAGATTCTCGCATCTGAGAAACTTGTGATCCCGTGGATCATTTAGTATTTGATT

TTCGGGAGACGCGAGTTTTTTTTTTTTAATATACATATAGTAACTTTTTCTTTTTTCTTTTAA
